# Multisensory feedback makes swimming circuits robust against spinal transection and enables terrestrial crawling in elongate fish

**DOI:** 10.1073/pnas.2422248122

**Published:** 2025-08-18

**Authors:** Kotaro Yasui, Astha Gupta, Qiyuan Fu, Shura Suzuki, Jeffrey Hainer, Laura Paez, Keegan Lutek, Jonathan Arreguit, Takeshi Kano, Emily M. Standen, Auke J. Ijspeert, Akio Ishiguro

**Affiliations:** ^a^Frontier Research Institute for Interdisciplinary Sciences, Tohoku University, Sendai 980-8578, Japan; ^b^Research Institute of Electrical Communication, Tohoku University, Sendai 980-8577, Japan; ^c^Biorobotics Laboratory, Institute of Bioengineering, École Polytechnique Fédérale de Lausanne, Lausanne CH-1015, Switzerland; ^d^Department of Biology, University of Ottawa, Ottawa, ON K1N 6N5, Canada; ^e^Department of Biology, Villanova University, Villanova, PA 19085; ^f^School of Systems Information Science, Future University Hakodate, Hakodate 041-8655, Japan

**Keywords:** amphibious locomotion, neuromechanics, computational model, pressure and stretch feedback, spinal cord transection

## Abstract

Vertebrate locomotion is due to the interplay of neural oscillators and sensory feedback loops in the spinal cord that interact with the body and the environment. Here, we study these circuits with a focus on undulatory locomotion as produced by elongated fish such as eels and lampreys. We address three questions: i) How do proprioception (stretch feedback) and exteroception (pressure on skin) interact with local oscillators to generate stable swimming patterns? ii) Can these feedback loops also contribute to dry ground locomotion? iii) Can they explain the remarkable robustness of eels against spinal cord transections? To address these questions, we developed abstract models of the locomotion circuits based on coupled phase oscillators, local stretch and pressure feedback loops, and simulated muscle models that were tested both in simulation and with a real undulatory robot. We also performed swimming experiments with eels before and after spinal cord transections. We found that stretch and pressure feedback work well together in swimming, as they contribute to rapid pattern generation and can, in principle, both replace direct couplings between oscillators. Interestingly, the swimming controllers could generate good ground locomotion when placed in an arena with pegs. For ground locomotion, the stretch feedback is more beneficial than pressure feedback. Finally, our models could replicate the remarkable ability of eels to keep swimming shortly after a full spinal cord transection. We found that stretch feedback and the ability of oscillators to spontaneously oscillate are likely explanations for keeping the neural oscillators active and coordinated below the transection.

## Significance

Despite a large diversity of morphologies and modes of locomotion, all vertebrate animals share locomotion controllers with the same general architecture. This work investigates how animal, simulation, and robot experiments can contribute to reverse-engineer the principles underlying these neural circuits. We highlight the potential role of local sensory feedback loops in generating swimming patterns. We show that circuits for swimming could contribute to dry ground locomotion when pushing against objects, potentially facilitating the transition from water to land during evolution. We show that stretch feedback and spontaneous oscillations could potentially explain the remarkable robustness of eels against spinal transections, a robustness that is unfortunately not observed in amphibians and mammals.

Undulatory locomotion is one of the most ubiquitous locomotor patterns in animals. For example, limbless locomotors such as nematodes (1), eels (2), and snakes (3) bend their flexible body in the lateral direction and propagate the body waves anterior–posteriorly to propel themselves forward. This locomotor mode is used by various animal species across invertebrates and vertebrates in diverse environments with different physical properties, including aquatic and terrestrial substrates (Table 1). These facts suggest that it may be driven by highly flexible locomotor control that can adapt to different physical interactions between the body and the environment. Therefore, understanding control mechanisms underlying undulatory locomotion could lead to extracting one of the universal principles of adaptive locomotor control in animals as well as provide insights into the transition from aquatic to terrestrial locomotion during evolution.

**Table 1.** Summary of the representative modeling and biological studies on undulatory locomotion in the literature

Animal	S,P	T	B,M,R	F,L	Summary	Reference
Lamprey	S	-	B	-	Studied edge cells using physiological, light microscopical, and electron microscopical techniques. Demonstrated depolarization of edge cells in lamprey’s stretched spinal cords.	Grillner (9)
Lamprey	-	T	B	F	Compared EMG activity of intact and spinal fish during swimming, as well as ventral root activity in in-vitro preparations. After spinal transection, lampreys could be induced to swim continuously by a light initial mechanical stimulation at the tail or dorsal fin. The proportion of burst activity and phase lag per segment remained unchanged in spinal animals compared to intact animals.	Wallén (26)
Lamprey	P	-	B	-	Verified and characterized the response of Touch (T) and Pressure (P) type dorsal cells, which were previously identified by Martin and Wickelgren (21). No evidence of nociception was found. Demonstrated different response latencies for T and P type cells.	Christenson (20)
Lamprey	S	-	B,M	-	Tested the effect of mechanically and rhythmically moving the extremities of an isolated spinal cord. The animal experiments showed that the frequencies of the CPG match those of the mechanical forcing within some range, demonstrating the role of mechanosensory feedback on entraining the CPG frequency. Models of coupled phase oscillators replicate the same effect.	Williams (27)
Lamprey	S	-	M	F	Developed a simplified neuromechanical simulation framework to study lamprey swimming behavior, integrating a CPG network with stretch feedback based on lamprey studies. The models were tested under perturbations like vortices and speed barrier.	Ekeberg (28)
Lamprey	S	-	M	F	Investigated the functional role of body curvature-based sensory feedback using a full Navier–Stokes model for fluid simulations. The effects on steady swimming, energetics, and kinematics were evaluated.	Hamlet (29)
Lamprey	S	T	M	F	Studied spinal injury in lampreys using a neuromechanical model. Partial or full recovery of swimming behavior was achieved by amplifying body curvature-based sensory feedback below the spinal lesion.	Hamlet (30)
*C*. *elegans*	S	-	B	F	Reported and characterized the proprioception-mediated response in *C*. *elegans* to perturbations in midbody bending amplitude. Used tools such as genetics, microfluidic and optogenetic perturbations, and optical neurophysiology to investigate the underlying neural circuit responsible for the response.	Ji (17)
*C*. *elegans*	S	-	B	F,L	Replicated swim-crawl transitions and locomotion in complex environments using proprioceptive responses. The neuromechanical control relied on feedback to generate undulation.	Boyle (19)
*C*. *elegans*	-	-	B,M	F,L	Conducted experimental and simulation-based investigations of swimming behavior in fluid-filled micropillar arrays. Simulations used a mechanical model incorporating hydrodynamic and contact interactions within the lattice.	Majmudar (31)
*C*. *elegans*	S	-	R	L	Studied and replicated swimming and crawling behavior. A bistable switch with hysteresis (Schmitt Trigger) combined with sensory feedback was used. The feedback signal encoded the weighted aggregate of bending across nine segments.	Boyle (32)
Leech	-	T	B	F	Compared behavioral and electrophysiological recordings of leeches with intact and severed ventral nerve cords. Active intersegmental coordination occurs in leeches with severed nerve cords, with the correct anterior-to-posterior progression of swimming activity always maintained.	Yu (13)
Leech	S	-	B	F	Reported the capability of ventral stretch receptors (VSR) in leeches to encode muscle contraction information during swimming via membrane potential oscillations. Modifying their activity, by injecting rhythmic current at different phases of the swim cycle, changes the intersegmental phase lags.	Cang (15)
Leech	S	-	B,M	F	Conducted experimental and simulation-based investigations on the adaptation of oscillation patterns in different environments. Muscle tension feedback at the spinal level was shown to be sufficient to produce adaptive patterns observed in leeches in both air and high-viscosity fluid.	Iwasaki (18)
Zebrafish	S	-	B	-	Identified intraspinal lateral proprioceptor (ILP) neurons in domes near the intervertebral disc. These neurons responded to stretch during lateral bending of the spinal cord, but not to compression. They project ascending contralateral axons for at least several spinal segments. In addition to their mechanosensing role, they act as inhibitory interneurons, likely terminating contralateral muscle activity that produces the bend.	Picton (12)
Salamander	S	-	B, M	F	Conducted experimental and simulation-based investigations of swimming and walking patterns in salamanders. Extended models based on the lamprey’s spinal cord, combined with a phasic drive, were used to replicate these patterns.	Bem (33)
Salamander	S	-	M	F,L	Explored the role of axial proprioceptive sensory feedback in salamander locomotion. The study used a neuromechanical model based on spiking neural networks, with detailed simulations of neuronal classes responsible for rhythm generation, for both in-vitro and in-vivo experiments in simulation.	Pazzaglia (34)
Snake	S	-	R	L	Developed torque control strategies to tackle environmental challenges, including obstacles. Three strategies were devised: two based on the robot’s local curvatures and a third utilizing joint velocities and torques as feedback. All controllers demonstrated effective adaptation in different environments.	Rollinson (35)
Snake	S	-	R	L	Devised control strategies for locomotion in environments with pegs and rocks. These strategies were based on shape-based compliance and admittance, using inferred obstructions from the robot’s shape during locomotion.	Wang (36)
Snake	S,P	-	R	L	Obstacle-aided locomotion was demonstrated using a controller that combined stretch-based curvature derivative control with reflex rules triggered by sensed pressure. The time taken and locomotion efficiency were analyzed.	Kano (37)
Snake	S,P	-	R	L	Demonstrated obstacle-aided locomotion where stretch feedback is integrated into control as the reference angle for each joint using a leader–follower scheme. Contact force feedback is utilized for resolving jams.	Liljeback (38)
U-Swimmer	P	T	M, R	F	Achieved oscillator entrainment for coordinated swimming using distributed, local pressure feedback. The feedback was tested across various configurations and under different disruptions. One condition lacks coupling between oscillators, corresponding to a multiple transection experiment.	Thandiackal (22)
U-Swimmer	S,P	T	B,M,R	F,L	Studied combined effects of stretch and pressure feedback. Combined feedback generates rapid swimming patterns. Additionally, stretch feedback enhances ground locomotion, and the combined model replicates eels’ ability to swim after spinal cord transection.	This paper

S; Stretch, P; Pressure, T; Transection, B; Biological study, M; Modeling study, R; Robotics study, F; Fluid, L; Land, U-swimmer; Undulatory swimmer.

An important aspect here is that locomotion emerges through complex and dynamic interactions between neural circuits, body mechanics, and the environment (4–6). Many neurobiological studies using various limbless animals have suggested key mechanisms for understanding such locomotor control systems (Table 1). In particular, studies on vertebrate undulatory swimmers such as lamprey and eels have greatly contributed to our understanding. The first key component of the locomotor circuits is the central pattern generators (CPGs) in the spinal cord, which are distributed neural networks that can generate basic rhythmic motor patterns for movement even in the absence of sensory inputs (7). This finding is pivotal. It has also been suggested that peripheral sensory feedback can play a role in generating coordinated rhythms between different body parts. For instance, intraspinal mechanoreceptive cells called edge cells in the lamprey (8–11), respond to the stretch of the spinal cord and act to terminate motor bursts by exciting ipsilateral neurons and inhibiting the contralateral ones. The same type of neurons are found in zebrafish (12). The role of stretch feedback is particularly important in invertebrates such as leeches (13–16) and worms (*Caenorhabditis elegans*) (17), as shown in different neuromechanical simulations (18, 19). In addition to such proprioceptive feedback, exteroceptive feedback, which arises from the interaction between the body and environment, has also been identified. In lamprey, such mechanoreceptors, called dorsal cells, are sensitive to touch and water pressure on the lateral sides of the body (20, 21). Further studies using simulation and robotic models have demonstrated that dorsal cell-like exteroceptive feedback mechanisms can entrain CPG activities to stabilize swimming patterns (22). Thus, in general, essential mechanisms are thought to be in the interplay between the CPGs and sensory feedback to generate coordinated locomotor movements against variable environmental situations. However, since most previous studies have focused on a single sensory feedback mechanism, how multiple sensory feedback mechanisms interact with CPGs to generate adaptive locomotor behaviors remains unclear. This is because it is still technically challenging to experimentally measure or manipulate the sensory-motor system in locomoting animals, especially when interventions involve multiple sensory systems.

To address this issue, we focus on amphibious locomotion of elongated fish, using an approach that combines neuromechanical modeling and experiments. It is known that some elongated-bodied fish, such as eels and ropefish, are able to move in terrestrial environments using axial body undulation (2, 23–25), similarly to aquatic swimming. Previous studies reported that there are kinematic differences between aquatic and terrestrial locomotion and suggested that elongated fish possibly exploit the heterogeneity of the terrain, such as pegs (23) on the ground, to obtain propulsive forces like terrestrial slithering snakes (2, 23). Accordingly, we expect that by studying the locomotor ability of elongated fish in aquatic and terrestrial environments, we can reveal how interaction between CPGs and multimodal sensory feedback allows animals to adapt their motion to different physical environments. By transecting the spinal cord, we can also address how continuity between body segments affects overall control and function. We hypothesize that (proprio- and exteroceptive) sensory feedback loops that are useful for (intact) swimming could also be useful for robustness against spinal cord lesions and for ground locomotion.

In this study, we explore the question of transitions from water to ground through the lens of robotics-inspired biology (39, 40), employing a synthetic approach that incorporates mathematical models, simulations, and robots. The advantage of our approach is that we can examine the biological hypotheses on the locomotor circuits systematically by changing the control configurations in the mathematical model (for instance, by systematically adding or removing components and testing their effect at different strength levels), taking the physical interaction between the body and environment into account. We hypothesized that the control circuits for aquatic swimming could also contribute to terrestrial locomotion in amphibious fish. In other words, this would mean that the transition from water to land for elongated animals during evolution would not have necessarily required the addition of new control circuits, but could have benefited from circuits that were readily available for swimming. To test this hypothesis, we designed neuromechanical and robotic models and evaluated whether the control circuits for aquatic swimming can provide locomotor ability in crawling on the ground with many obstacles. Specifically, we explored the combination of CPGs, stretch feedback, and pressure feedback in amphibious locomotion, and addressed the following questions: i) how does the combination of stretch and pressure feedback affect swimming? (for instance, in terms of stability of the locomotor patterns and time to reach steady-state regimes); ii) could these two types of feedback contribute to terrestrial locomotion in an arena with pegs?; and iii) could they also explain why eels are so robust against spinal cord lesions?

Through simulation and robot experiments, we found that stretch and pressure feedback (which had been studied only separately in the past) work well together in swimming and contribute to rapid pattern generation. For ground locomotion, the stretch feedback is beneficial, whereas pressure feedback alone did not contribute to forward locomotion. The stretch feedback can generate effective locomotor movement that exploits propulsive reaction forces from the pegs on the ground. Furthermore, we investigated the robustness of locomotion against spinal cord transections. We demonstrated that eels can swim shortly after a full spinal cord transection, replicating previous experiments in eels (41), lamprey (26), and leech (13, 14). With our models, we found that the stretch feedback plays an important role in coordinating the swimming motion between body parts above/below the transection. Indeed, combined with the ability of local oscillators to generate spontaneous oscillations without excitatory inputs, the stretch feedback leads to coordinated swimming patterns that closely resemble intact swimming, similarly to the eel experiments. Consequently, our proposed model may capture the possible common control circuits underlying aquatic and terrestrial undulatory locomotion and could pave the way for control design methodology for limbless bioinspired robots with high environmental adaptability.

## Related Work

Table 1 provides an overview of previous biological and modeling studies on the role of stretch and pressure feedback in undulatory locomotion. Most modeling studies have focused on stretch feedback during swimming and, to a lesser extent, during ground locomotion. In contrast, pressure feedback has been much less studied, except for our previous modeling and robotic study of undulatory swimming (22), as well as obstacle-based locomotion in snake robots (37, 42, 43). Modeling studies of robustness against spinal cord transections have been proposed based on stretch feedback (30) and on pressure feedback (22). While the individual benefits of pressure and stretch feedback have been demonstrated, the combined effects of the two remain unexplored. Since locomotion is a highly nonlinear phenomenon, combining two different feedback loops could, in principle, lead to unexpected behavior, making it essential to investigate their combined effects. Our work investigates stretch and pressure feedback, swimming and obstacle-based terrestrial locomotion, as well as robustness against spinal cord transection in a single study. In the next sections, we will compare in more details our findings with previous work.

## Modeling

### Neuromechanical Simulation and Robot.

**Fig. 1.**
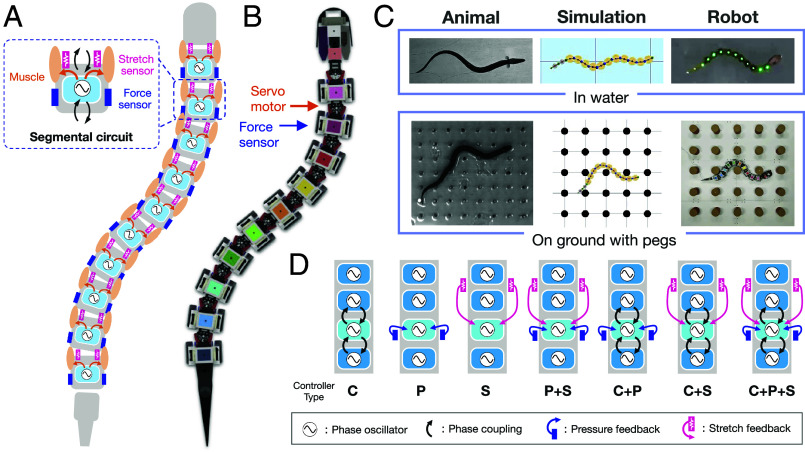
Overview of our neuromechanical modeling approach. (*A*) Schematic of the neuromechanical model of elongated fish. The body consists of rigid body segments connected by left and right antagonistic muscles. Each segment has a local circuit with an oscillator and a pair of lateral pressure force sensors and stretch sensors. (*B*) Overview of the developed robot (CAD image) to implement hypothesized control circuits and evaluate locomotor performance in aquatic and terrestrial environments. (*C*) Our synthetic approach to study the locomotor circuits for swimming and crawling. The image of the ropefish on the ground with pegs borrowed from figure 2D of Ward et al. (23) with permission. (*D*) Tested different control configurations in our model. C; central phase coupling between the oscillators, P; pressure force feedback, S; stretch feedback. Note that we illustrated the signal connections for the single segmental oscillator, which are highlighted in cyan.

We constructed a neuromechanical model of elongated fish and tested it using computer simulations and robots to investigate the ability of CPG-based neural circuits with pressure and stretch feedback in amphibious locomotion. The body was modeled as a planar chain of 10 rigid segments connected by rotational joints (Fig. 1*A*). At each joint, viscoelastic body deformation is generated by signals from the segmental circuit that coordinate the left–right alternated activations of a pair of antagonist muscles (*Materials and Methods*). We also assumed that each local body segment possesses stretch and pressure sensors at the left and right sides of the body. Since the physical interactions between the body and environment affect the resultant locomotor movements and can only be approximated in simulation, we used a real physical robot to validate the simulation results (Fig. 1 *B* and *C*). Specifically, we used the undulatory swimming robot named AgnathaX, which was developed in our previous work (22), for swimming experiments in water. Furthermore, we developed more robust pressure sensors in this study for use in crawling experiments on the ground.

### Design of the Neural Circuits Based on Multimodal Local Sensory Feedback.

Similarly to previous modeling work (27, 30, 44, 45), we model the locomotor circuits using abstract models made of coupled phase oscillators. We assume that the timing of muscle activation ($M_{i}$) at each body segment is determined by the local activation of the segmental neural circuit, which is described by the phase of oscillator ($\phi_{i}$):

[1] $$\begin{array}{c} M_{i}=\;\cos\phi_{i}. \end{array}$$

$M_{i}$ controls the internal torque and therefore induces bending to either side of the body. This model assumes a fixed left–right intrasegmental coordination, in which the muscle pair operates in antiphase (contracting on the right when $M_{i}>0$ and on the left when $M_{i}<0$).

We designed the time evolution of each local oscillator using the following Eqs. **2**–**5**:

[2] $${\dot{\phi }}_{i}\;\;\;\;=2\pi f_{i}+g_{c,i}+g_{p,i}+g_{s,i},$$

[3] $$g_{c,i}=\sigma_{c}\{\sin(\phi_{i-1}-\phi_{i}-\psi )+\sin(\phi_{i+1}-\phi_{i}+\psi )\},$$

[4] $$g_{p,i}=\sigma_{p}{\hat{F}}_{i}\cos\phi_{i},$$

[5] $$g_{s,i}=-\sigma_{s}\theta_{i-1}\sin\phi_{i},$$

where $f_{i}$ is the intrinsic frequency of the oscillator, $g_{c,i}$ is neural coupling between the adjacent oscillators (nearest neighbor coupling), and $g_{p,i}$ is the pressure sensor feedback originally proposed in our previous study for aquatic swimming (22) and extended for terrestrial crawling. $g_{s,i}$ is the stretch sensor feedback proposed in this study and $\theta_{i-1}$ denotes the bending angle (i.e., local curvature) of the nearest anterior segment. In this model, the intrinsic oscillator frequency ($f_{i}$) is assumed to be the same for all oscillators, except for the transection experiments. In biological experiments, the intrinsic frequency of the local oscillators tends to increase with the activation of descending drive (or the application of excitatory chemicals for isolated spinal cords). In many models (30, 45), the intrinsic frequency is assumed to be zero when descending drive is absent. Here, we hypothesize that neural oscillators in eels can spontaneously produce oscillations (i.e., $f_{i}>0$ even without descending drive), and the effect of this hypothesis will be tested in the transection experiments. Note that the resulting frequencies observed during locomotion can be different from the intrinsic frequencies due to the effect of sensory feedback signals and interoscillator couplings.

Similarly, the phase lags between oscillators will be influenced by the interoscillator couplings and by sensory signals when interacting with the environment. In Eq. **3**, $\sigma_{c}$ defines coupling weights, and *ψ* denotes a central phase lag bias with the adjacent oscillators, which influences the number of body waves. Eq. **4** describes the feedback mechanism based on the local entrainment of phase $\phi_{i}$ by the local pressure force ${\hat{F}}_{i}$ with a feedback gain $\sigma_{p}$. The reasoning behind the equation can be found in ref. 22. In short, it implements couplings between the (periodic) forces applied on the skin and the local phases, which correspond to ipsilateral excitatory and contralateral inhibitory connections from pressure sensors to the local oscillators like in the lamprey (22). Note that ${\hat{F}}_{i}$ represents the sensory information of the net pressure force, which is the difference in the forces exerted on the body segment from the left and right sides, with its magnitude saturating at a threshold value $F_{\mathrm{th}}$. This sensory saturation mechanism is introduced in this study so that the pressure feedback cannot reach excessive values (e.g., when pushing hard against a peg), which were found to be detrimental in preliminary tests. In aquatic swimming, ${\hat{F}}_{i}$ is assumed to be the net hydrodynamic force, which is proportional to the square of the velocity of the body segment; similarly to Ekeberg (46). See *SI Appendix* and previous swimming results using this force feedback control in Thandiackal et al. (22).

The stretch feedback (Eq. **5**) was designed based on a control principle derived in the work on undulatory locomotion of snake-like robots (47). Date and Takita (47) used a continuum model of a snake-like slender body and mathematically showed that transmitting a traveling wave along the body with posterior joints “copying” the movements of anterior joints is energetically optimal for slithering locomotion on the ground, while the control scheme is quite simple. Motivated by their work, we hypothesized that a similar control principle may be exploited in the undulatory locomotion of elongated fish, and such mechanisms can explain locomotor ability in both aquatic and terrestrial locomotion. In Eq. **5**, $\theta_{i-1}$ is the bending angle (i.e., local curvature) of the nearest anterior segment and $\sigma_{s}$ is the feedback gain. Note that we used the body bending angle as an alternative to the differences in the stretch sensor inputs from the left and right muscles along the body segment. The proposed stretch feedback works as follows: when the *i*th segment is not bending (i.e., the oscillator phase $\phi_{i}$ is around $\frac{1}{2}\pi$ and $\frac{3}{2}\pi$), the sensory input of large body bending at the *i-1*th body segment modulates the *i*th oscillator phase so as to bend in the same direction of the anterior segment. Additionally, we also explored different topologies for the stretch feedback with different projections up and down the chain (see Eq. **11**, *Additional Simulation Experiments to Explore Different Topologies for the Stretch Feedback*). These experiments showed that other topologies could also contribute to swimming (*SI Appendix*, Fig. S3 and Movie S5), but confirmed that the feedback mechanism we chose (Eq. **5**) was the best for also contributing to terrestrial locomotion (*SI Appendix*, Fig. S4).

### Definition of Tested Controller Types.

To investigate the contribution of each circuit component (Eq. **2**) to locomotor performance, we evaluated different controller types in both aquatic swimming and terrestrial crawling. Specifically, we examined seven types of controllers (Fig. 1*D*), encompassing all possible combinations of three circuit components: central phase coupling between adjacent oscillators (“C”), pressure sensor feedback (“P”), and stretch sensor feedback (“S”). Note that we assumed intrinsic oscillation for each segmental circuit ($f_{i}>0$) in all controller types and denoted the controllers with a single circuit component as C, P, S, and those with combinations of two or three components as “P+S,” “C+P,” “C+S,” and “C+P+S.”

## Results

### Pressure and Stretch Feedback Contribute to Rapid and Stable Pattern Generation in Swimming.

We conducted swimming experiments in simulations and with the robot to understand how our proposed stretch and pressure feedback work under various configurations in our CPG-based circuits. In simulations, we measured 30 s of swimming for each controller type. We tested each controller type 20 and five times in the simulations and the robot experiments, respectively, all starting with a straight pose. Note that the initial oscillator phases were randomized in all trials to test the controller’s ability to robustly establish swimming patterns in the body.

**Fig. 2.**
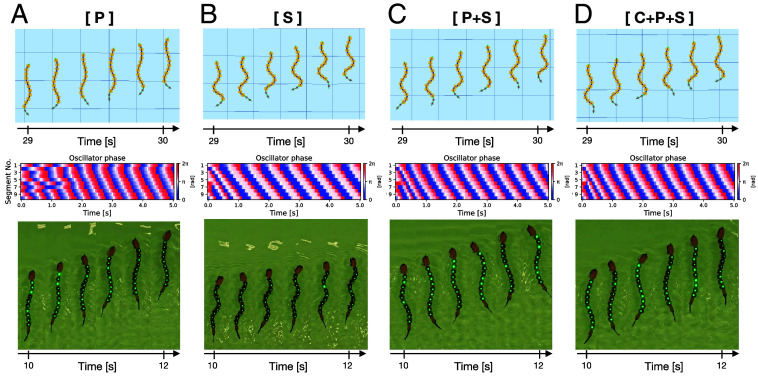
Representative examples of the swimming generated using different controller types in simulations and with robots: (*A*) pressure feedback only (type P), (*B*) stretch feedback only (type S), (*C*) combination of pressure feedback and stretch feedback (type P+S), (*D*) combination of central phase coupling, pressure feedback, and stretch feedback (type C+P+S). Panels in the second row are the spatiotemporal plots of the oscillator phases in simulations. Note that the intrinsic oscillator frequency ($f_{i}$) was set to be 1.5 Hz and 0.75 Hz for simulations and robots, respectively.

Fig. 2 shows representative snapshots of the swimming under different controller types and their spatiotemporal plots of the oscillator phases. In every controller type (C, P, S, P+S, C+P, C+S, C+P+S), a stable swimming motion, where the traveling wave pattern propagated from the head to tail, was generated (see Movie S1 for the results of types C, C+P, C+S). In particular, it should be noted that for the purely sensory-driven controllers (type P, S, and P+S; Fig. 2*A*–*C*), no coordination between oscillators was centrally defined in advance because the segmental circuits provided no intrinsic intersegmental coupling (as in the controller type C). Therefore, the swimming patterns produced by such sensory-driven controllers are nontrivial and emergent phenomena. In addition, the results indicate that the proposed stretch feedback does not conflict with central phase couplings and pressure feedback to produce coordinated swimming motions.

**Fig. 3.**
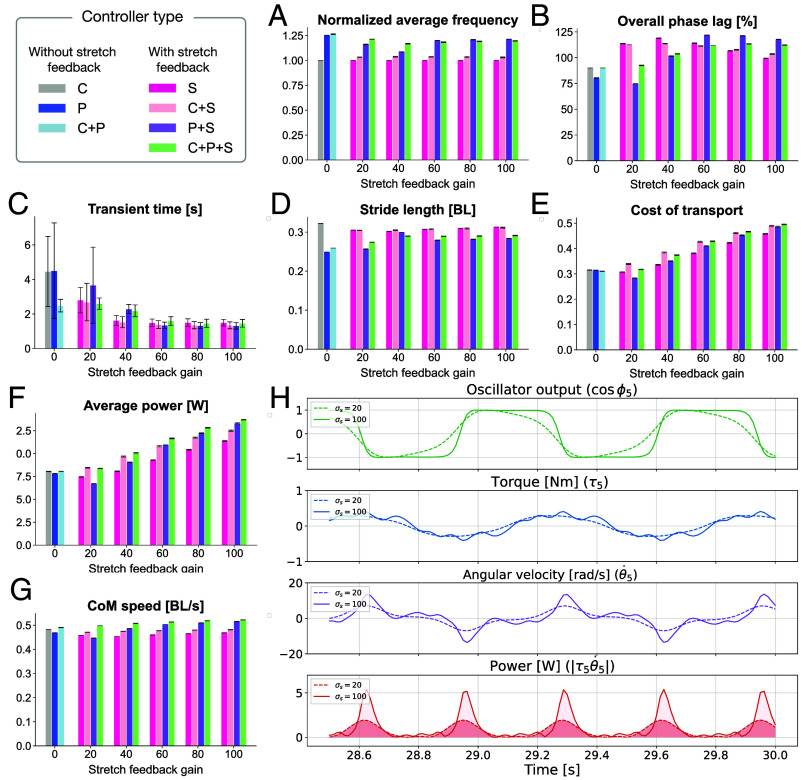
Comparison of generated swimming patterns and their performance with different controller types in simulations: (*A*) swimming frequency normalized by the intrinsic oscillator frequency (1.5 Hz), (*B*) overall phase lag along the body from the head to tail, (*C*) transient time required to reach steady-state swimming, (*D*) stride length per cycle normalized by body length (BL), (*E*) mechanical cost of transport (CoT), (*F*) average power, (*G*) center of mass (CoM) speed normalized by body length, (*H*) Representative time series data from the middle (5th) segment when using controller type S, which implements stretch feedback only. Two cases with different feedback gains are compared: $\sigma_{s}=20$ (dashed line) and $\sigma_{s}=100$ (solid line). From top to bottom, the plots show the local oscillator output, generated joint torque, angular velocity, and the absolute value of mechanical power. Error bars in (*A*–*G*) indicate the SD. All evaluation metrics, except for panels *C* and *H*, are calculated based on steady-state motion observed during the final 3 s (27 to 30 s) of the simulation experiment.

To capture basic characteristics of resulting swimming patterns depending on different controller types, we measured swimming frequency and intersegmental phase lag along the body (i.e., “overall phase lag”; see *Materials and Methods*). We found that the stretch feedback controller (type S) maintained swimming frequency at the level of the intrinsic oscillator frequency ($f_{i}$ in Eq. **2**), while the pressure feedback controller (type P) worked to increase it (Fig. 3*A*). Furthermore, when these two sensory feedbacks were combined (types P+S, C+P+S), the stretch feedback also tended to maintain the faster oscillator rhythms established by the pressure feedback. Regarding overall phase lag along the body, two opposing effects of stretch feedback were observed depending on the controller combinations (Fig. 3*B*). Specifically, when the controller is solely based on stretch feedback (type S), the overall phase lag tended to decrease as the feedback gain increased. In contrast, when the stretch feedback was combined with pressure feedback (types P+S, C+P+S), the higher stretch feedback gain resulted in an increase in the overall phase lag, which means an increase in the wave number.

To compare the swimming performance according to the different controller types, we quantitatively evaluated the transient time to reach the steady-state swimming, the stride length, and the mechanical CoT (Fig. 3*C*–*E*; see the definition of each metric in the Materials and Methods). The most important finding was that stretch feedback led to faster convergence to steady-state swimming (Fig. 3*C*). Our proposed stretch feedback controller (type S) enabled convergence to stable patterns within a few cycles from random initial phases. Notably, the transient time of the centrally phase-coupled controller (type C) and pressure feedback controller (type P) tended to shorten when they were combined with stronger stretch feedback (types C+S, P+S, and C+P+S). Additionally, Fig. 3*D* showed that increasing the stretch feedback gain tends to have a positive effect on increasing the stride length of the swimming for pressure-feedback-based controllers (types P+S, C+P+S), whereas the stride lengths of the stretch-feedback-based swimming (types S, C+S) were as high as the centrally phase-coupled controller (type C). Finally, we observed that the higher stretch feedback strength resulted in higher mechanical costs of transport (Fig. 3*E*). This is primarily due to an increase in mechanical power (Fig. 3*F*) and is not affected by swimming speed (Fig. 3*G*). The following mechanism is likely to explain this phenomenon (Fig. 3*H*): In the proposed control scheme, the stretch feedback tends to generate oscillator output signals that are more “square-like” than the sinusoidal signals without feedback. Indeed, the stretch feedback keeps the oscillator phase near the phase of maximum bending (i.e., $\phi_{i}=0,\pi$) as long as the anterior segment is bending in the same direction. However, once the anterior segment begins to bend in the opposite direction, the stretch feedback accelerates the phase, pulling it toward the maximum bending phase on the opposite side. As the stretch feedback gain increases, this effect intensifies, leading to greater accelerations and decelerations in the angular velocity of the body bending motion. This increased variability in angular velocity results in higher instantaneous mechanical power, thereby increasing the mechanical CoT.

### Stretch Feedback in Swimming Circuits Enables Obstacle-Aided Terrestrial Locomotion.

Next, we tested our hypothesis that the control circuits for aquatic swimming could also contribute to terrestrial locomotion. It is considered that elongated fish can move across uneven terrestrial environments in nature because they effectively obtain reaction forces from the contact between their bodies and the irregularities of the ground (23, 25). Consequently, we set up a dry arena with circular pegs arranged at uniform distances in a grid, representing a terrain that provides heterogeneous physical interactions with the fish body.

Furthermore, to focus on propulsion through the use of obstacles (as opposed to through interactions with the flat ground), we assumed isotropic friction on the ventral surface of the body. In other words, the robot/simulation was not able to move forward by itself on purely flat ground, similarly to undulatory fishes and unlike snakes (which have directional friction). In both simulation and robot experiments, the body was initially placed in a diagonal orientation with the head toward the center of the lattice with pegs, and the initial oscillator phases were randomized. We tested each controller type 20 and five times in the simulations and the robot experiments, respectively. Each trial was judged as a successful traverse if the head reached any border of the peg area (see *Materials and Methods* for the details of the experimental setup).

**Fig. 4.**
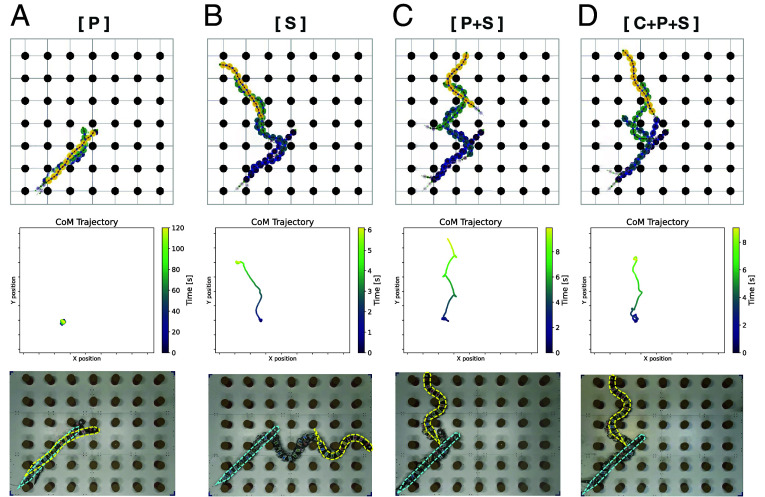
Representative examples of crawling locomotion on the ground with pegs using different controller types. The *Top* and *Bottom* panels are the snapshots of the simulation and robots, respectively. The *Middle* panels are the trajectory of the CoM in simulations. The initial and final positions of the robot are shown with cyan and yellow colors, respectively. Note that the intrinsic oscillator frequency ($f_{i}$) was set to be 0.5 Hz and 0.25 Hz for simulations and robots, respectively. Tested controller types are (*A*) pressure feedback only (type P), (*B*) stretch feedback only (type S), (*C*) combination of pressure and stretch feedback (type P+S), (*D*) combination of central phase coupling, pressure feedback, and stretch feedback (type C+P+S).

Fig. 4 displays representative snapshots and the trajectory of the CoM during crawling locomotion on the ground under different controller types. We observed that while the pressure feedback controller (type P; Fig. 4*A*) can hardly propel from the initial position, the stretch feedback controller (type S; Fig. 4*B*) and combined controllers with stretch feedback (type P+S and C+P+S, Fig. 4 *C* and *D*) could immediately generate a body undulation with a large curvature, enabling effective propulsion (Movie S2).

**Fig. 5.**
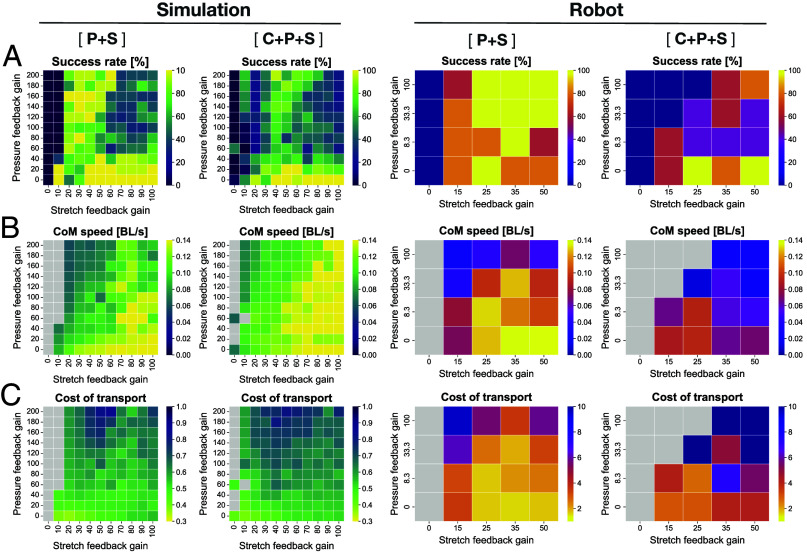
Comparison of the locomotor performance on the ground with pegs using different feedback gains for pressure and stretch feedback. The *Left* panels are the simulation results, while the *Right* panels are the robot results. (*A*) Rate of successful traversal, (*B*) Average CoM speed normalized by body length (BL), and (*C*) Mechanical CoT. (*B* and *C*) only include successful trials and cells shaded in gray indicate that there were no successful trials. Note that the intrinsic oscillator frequency ($f_{i}$) was set to be 0.5 Hz and 0.25 Hz for simulations and robots, respectively. Accordingly, the central phase coupling weight ($\sigma_{c}$) and sensory feedback gains for the stretch ($\sigma_{s}$) and pressure ($\sigma_{p}$) for the robots were half of those in simulations.

To further investigate how the strength of pressure and stretch feedback affect locomotor performance on the ground with pegs, we evaluated the rate of successful traverse, speed, and energetic efficiency by varying the sensory feedback gains (Fig. 5). First, we confirmed that with the pressure feedback alone (type P), regardless of its feedback gain, it was quite difficult to traverse the peg environment, and even adding central phase coupling (type C+P) did not change the outcome (Fig. 5*A*). In contrast, the stretch feedback controller alone (type S) could achieve a smooth traverse across the peg environment with a high success rate. Interestingly, when pressure feedback and stretch feedback are combined (type P+S and C+P+S), the proposed controller still achieves a relatively high success rate in traversing the peg environment. This suggests that, although pressure feedback alone does not contribute to effective propulsion, it does not necessarily impair locomotor performance when combined with stretch feedback, and that certain balances of stretch and pressure feedback gains may enable successful traversal. Fig. 5 *B* and *C* display the CoM speed and the mechanical CoT for the trials of successful traversal, respectively. We found that i) as the pressure feedback gain increases, both CoM speed and the CoT tend to worsen, and ii) as the stretch feedback gain increases, CoM speed tends to improve while CoT shows a slight increase. The simulation and robot experiments tend to be in good agreement except for the higher success rates for the robot with higher levels of stretch and pressure gains, which are likely due to sim-to-real mismatches (e.g., differences of the exact geometries of the robot segments and/or the interaction forces with the pegs and the ground). Finally, it is worth mentioning that the centrally phase-coupled controller (type C) was able to traverse the peg environment in a few trials, depending on the initial conditions (see the case of C+P+S in simulations when both the pressure and stretch feedback gains are set to zero in Fig. 5. The success rates were low but not zero). This suggests that obstacle-based locomotion is, in principle, possible even with open-loop control thanks to the compliance of the body (in our case provided by the simulated muscles), as also shown by Wang et al. (48).

### Multimodal Sensory Feedback Contributes to Robust Swimming After Spinal Cord Transection in Elongated Fish.

**Fig. 6.**
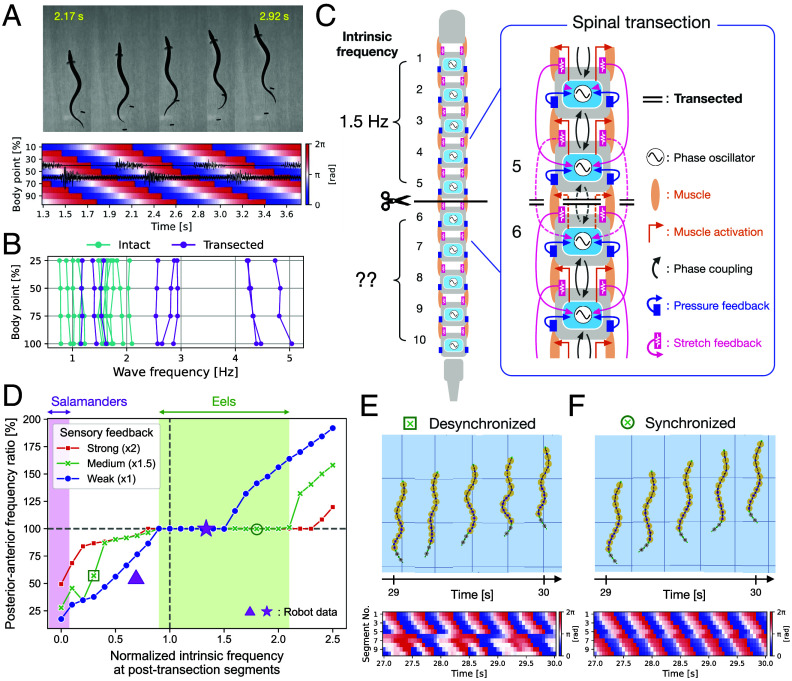
Frequency-locked swimming after spinal transection in eels and our model. (*A*) Eel swimming after the spinal cord transection at the middle of the body (approximately 50% body length) (Movie S3). The top figure shows representative snapshots of the swimming, and the bottom figure shows the spatiotemporal plots of the kinematic phase overlaid with the EMG signals from the right side of the body (black lines). (*B*) Body wave frequency at the different body points (25, 50, 75, and 100% of the length from head to tail). The data were measured based on kinematics from both intact individuals (cyan) and spinal transected ones (magenta). Each line represents one trial data. (*C*) Schematic of the spinal cord transection experiments in simulation. (*D*) Evaluation of the synchronization level of the swimming frequencies between the segments above/below the spinal transection using our model. Controller type C+P+S was used, and different strengths of sensory feedback were tested. The points marked with a triangle and star represent robot data (*SI Appendix* and Movie S4). (*E* and *F*) Examples of desynchronized and synchronized swimming, respectively. Snapshots of the swimming and spatiotemporal plots of the oscillator phases when the intrinsic oscillator frequency at the segments below transection was low (30%) and high (180%), respectively. The magenta-colored segments indicate the section below the spinal transection. In the first case, there is no proper synchronization below the transection, whereas there is in the second case (Movie S3). The same experiment was replicated with the robot with similar results (Movie S4).

To examine the robust swimming ability of elongated fish, we first performed spinal cord transection at the middle of the body (approximately 50% body length) using eels (*Anguilla rostrata*) and measured both the kinematics and muscle activities above/below the transection (see experimental details in *Materials and Methods*). Representative swimming data of the transected eel are shown in Fig. 6*A* (Movie S3). Our results matched those reported in a previous study (41). Transected eels exhibited swimming with undulatory body waves propagating from head to tail. It should be noted that not only the kinematic body wave but also the timing of muscle activation propagated across the transection site despite no nervous connection. This indicates that swimming movement of the body below the transection was coordinated through active motor control above and below the transection, not just passive deformation. Furthermore, we confirmed that body wave frequencies above and below the transection were similar (Fig. 6*B*). These results suggest that some mechanisms exist to synchronize the overall swimming frequency across the body, even if descending excitatory input to the segmental CPG circuit below the transection is lost.

To explore the possible control mechanisms that enable spinal-transected eels to generate coordinated and frequency-locked swimming, we performed spinal transection of our proposed model and observed the swimming in simulations (Fig. 6*C*–*F*). As an experimental setup, we used the all-combined controller (type C+P+S) for each segmental circuit and removed the neural and stretch feedback connections at the middle of the body (between the 5th and 6th segments, Fig. 6*C*). More specifically, the central phase coupling between the 5th and 6th oscillators and the stretch feedback connection to the 6th oscillator were removed. Also, the left and right muscles between the 5th and 6th segments were set to be completely passive, assuming that the muscles around the transected part lose active motor control. Note that we tested 20 times in the simulations for all experiments in this section with the initial oscillator phases randomized, starting from straight posture.

To evaluate the ability to synchronize the swimming frequency across the transected part, the intrinsic oscillator frequency ($f_{i}$ in Eq. **2**) for the posterior segments was varied by steps of 0.1 from 0.0 to 2.5 times higher than that of the anterior segments (1.5 Hz). These different levels were tested because we do not know the intrinsic frequencies of local oscillators when they do not receive a descending drive. Different strengths of sensory feedback were also tested by scaling both the stretch and pressure sensory feedback gains by a constant factor. Simulation experiments with the all-combined controller (type C+P+S, Fig. 6*D*) revealed that frequency-locked swimming can be achieved under the spinal transection and that higher sensory feedback gain allows for greater tolerance of intrinsic frequency discrepancies and enables synchronization. We also found that synchronization of swimming frequencies tends to be more easily achieved when the intrinsic oscillator frequency of the body posterior to the transection is higher than that of the anterior body. For instance, Fig. 6 *E* and *F* shows the snapshots and spatiotemporal plots of the oscillator phases during swimming at the medium strength of sensory feedback when the intrinsic oscillator frequency at the segments below transection was low (30%) and high (180%), respectively. The first case represents an example of desynchronized swimming, where the frequencies of the oscillators below the transection do not match those above the transection (Fig. 6*E*), whereas the second case represents an example of synchronized swimming (Fig. 6*F*), where the sensory feedback ensures a same resulting frequency for all oscillators (Movie S3 in simulation, and Movie S4 for a similar experiment with the real robot).

**Fig. 7.**
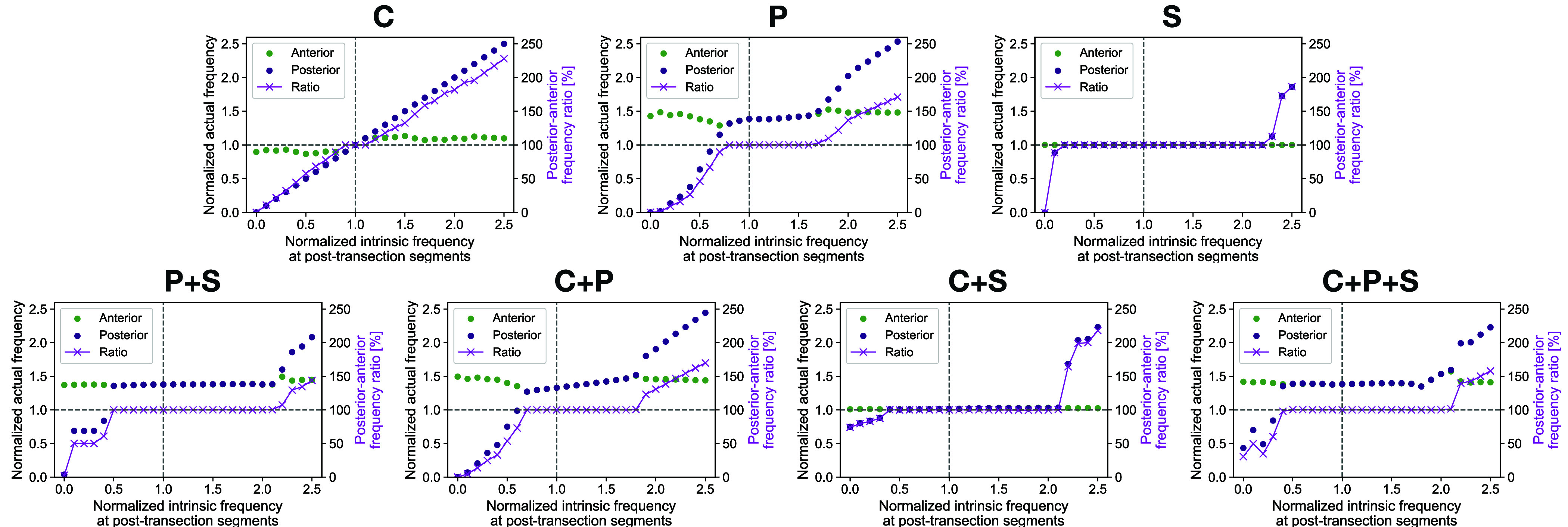
Comparison of frequency locking ability during swimming in simulated spinal-transected fish, using different controller types in our model. Spinal transection was simulated in the same manner shown in Fig. 6*C*. Each panel shows the actual frequencies in the body section above (green) and below (purple) the transection site, normalized by 1.5 Hz, and the synchronization level (magenta) evaluated by taking the actual frequency ratio between the anterior and posterior sections. Horizontal axes indicate the intrinsic oscillator frequency at the posttransection segments which is normalized by that of the anterior segments (1.5 Hz).

Similarly, we further examined the source of frequency-locked swimming after spinal transection in our model, using all the possible controller types. Specifically, here we assumed that all segmental circuits in a given trial had an identical controller type. The simulation results in Fig. 7 revealed that the purely pressure-feedback-based controller (type P) could synchronize the frequency above/below the transection (when the intrinsic frequency at the posttransection segments was from 80 to 160%). It was also found that the purely stretch-feedback-based controller (type S) was able to consistently produce synchronized swimming, except when the intrinsic frequency of the posterior part below the transection was close to zero or too high (more than 230% of the anterior segments). Note that the actual frequency of the synchronized swimming produced by the stretch feedback was the same as the intrinsic frequency of the anterior segments (see panel S in Fig. 7). In other words, the oscillators in the posterior segments below the transection were entrained to the frequency of the anterior segments, regardless of whether the anterior frequency was higher or lower. This synchronization is likely achieved due to the asymmetric control structure of our proposed stretch feedback, which references only the bending motion of the anterior segments, and the passive motion at the transection site transmitting the rhythm from the anterior to the posterior segments. Although stretch feedback appears to be most robust on its own, more interestingly, when sensory feedbacks were combined (i.e., type P+S), including central phase coupling (i.e., types C+P, C+S, C+P+S), this remarkable frequency-locking capability was maintained across a broad range, at least when the posterior intrinsic frequency was between 70 and 180% of the anterior frequency. These results suggest that both pressure and stretch sensory feedback can contribute to the generation of frequency-locked swimming and that the presence of at least two out of the three control components (C, P, S) allows adaptation to significant changes in the intrinsic frequency of the posterior part below the spinal transection.

Our results suggest that multimodal sensory inputs can be redundant without overly compromising function. More simply, the frequency-locked swimming in transected eels can be explained by two main characteristics: the strengths of the sensory feedback [as also proposed by Hamlet et al. (30) for stretch feedback] and the ability of neural oscillators to generate spontaneous oscillations at nonzero intrinsic frequencies, even in the absence of descending excitatory inputs from higher centers. It is likely that other undulatory swimmers that cannot swim immediately after a transection (like salamanders, in which the part of the body below transection remains inactive) lack one of these two components and therefore typically exhibit nonsynchronized swimming as observed on the left part of Fig. 6*D*.

## Discussion

The main contributions of this study are i) the proposal of a CPG circuit combining stretch and pressure sensory feedback, which functions in both aquatic swimming and terrestrial crawling locomotion; and ii) the prediction, derived from the proposed model, that the mechanism enabling robust swimming after spinal cord transection in elongated fish may be explained by local sensory feedback and the spontaneous oscillation of the local CPG circuits. In addition, multimodal sensory feedback increases redundancy/resilience in the system without limiting function. These findings were made possible through a robotics-inspired biology approach using neuromechanical models of simulations and robots to test neurological alterations that are otherwise difficult or impossible to perform in a living system. The predictions and hypotheses suggested by our abstract model will hopefully guide neurophysiological experiments and contribute to uncovering more detailed mechanisms in animals.

We proposed a stretch feedback mechanism for CPG-based controller, inspired by the control law derived for snake-like slithering locomotion (47), where the local segmental circuit refers to lateral bending of the anterior segment and adjusts the oscillator phase to bend in the same direction as the anterior segment (Eq. **5**). In contrast, previous studies on swimming circuits have discussed stretch feedback that works to prevent excessive local bending (11, 46), and recently, stretch feedback that transmits signals from posterior to anterior segments was reported in zebrafish (12). Therefore, our proposed stretch feedback works to enhance and transmit local bending and suggests a possible sensory feedback pathway from anterior to posterior segments. As a neural circuit counterpart, our model predicts that stretch receptors on one side of the body may have descending axons that excite the contralateral interneurons and motoneurons caudally in the spinal cord. More interestingly, through our additional exploration of different topologies in the stretch feedback mechanism (*SI Appendix*, Fig. S3 and Movie S5), we found that zebrafish-like posterior-to-anterior projection of the stretch feedback (12) can also lead to swimming (*SI Appendix*, Fig. S3*D*) but not to obstacle-aided crawling in our control scheme (*SI Appendix*, Fig. S4). These results suggest that our mathematical framework for the stretch feedback (Eq. **11**) may have generality in describing stretch sensory feedback mechanisms in undulatory swimming and also suggest that anterior-to-posterior projection of the stretch feedback may be essential to explain not only swimming but also terrestrial locomotor ability in elongated fish. Although our study assumed only local connections for central phase coupling (Eq. **3**) and pressure feedback (Eq. **4**), interneurons in the spinal cord have been reported to project across multiple segments in various animal species, including lampreys (49). Therefore, effects of nonlocal neuronal connections, such as long-range coupling with distant segmental circuits, on generation of locomotor patterns remain important topics for further investigation (see ref. 50 for some first results with longer-range stretch feedback).

Our experimental results for swimming also demonstrated that the proposed stretch feedback notably contributes to rapid convergence of the swimming pattern (Fig. 3*C*) and has a positive effect on increasing stride length when combined with pressure feedback (Fig. 3*D*). On the other hand, we found that high stretch feedback gain could reduce energetic efficiency (Fig. 3*E*). Thus, a meaningful next hypothesis to test would be whether integrating our proposed stretch feedback with the alternative one that works to prevent excessive local bending (i.e., to combine different types of stretch feedback projections) could self-organize a swimming pattern that also meets requirements for energetic efficiency (51) and robustness against perturbations (28).

This modeling study has some implications for terrestrial locomotor ability of elongated fish driven by undulatory body waves. We showed that owing to the proposed stretch feedback, the swimming circuits could also generate effective terrestrial crawling locomotion exploiting reaction forces from the pegs (Fig. 4*B*–*D*), while the pressure feedback alone could not benefit from obstacles on the ground (Fig. 4*A*). These different behavioral consequences may be explained by two factors: i) unlike aquatic swimming (22), where the whole body receives pressure forces, contact points with pegs along the body are too sparse, making it difficult to establish intersegmental coordination through pressure feedback. ii) The proposed locomotor circuits are based on rhythmic movements driven by CPGs, which often cause the body to lose contact with pegs before sufficient force develops at the body–peg interaction. These factors prevent the generation of snake-like obstacle-aided locomotion, highlighting a limitation of periodic oscillator-based locomotor control. Indeed, in previous models that successfully replicated snake-like obstacle-aided locomotion (42, 43), rhythmic oscillations are not assumed endogenous. Instead, they suggested a local sensory feedback mechanism combined with a reflexive curvature derivative control (47), which flexibly controls body bending in response to the interactions between the body and the environment. Therefore, there may be differences in motor control mechanisms between elongated fish, which rely on periodic CPG circuits, and snakes, which have adapted to terrestrial environments and more actively exploit terrain irregularities to achieve effective propulsion. Further advancing neurophysiological and anatomical studies on potential homologous mechanisms (52, 53) that enable pressure and stretch feedback, such as dorsal cells and edge cells known in elongated fish, across various animal species would be important for understanding both species-specific differences and common principles. And a final point concerning ground locomotion, is that the viscoelastic properties of the body play an important role in undulatory locomotion. Indeed, obstacle-based locomotion can in principle be obtained with open-loop control (i.e., without sensory feedback), as demonstrated by Wang and colleagues using an innovative compliant robot with bilateral actuation (48). The interplay of passive and active properties of the musculoskeletal system together with control loops remains a topic that can be investigated more deeply.

Our model also provides new insights into the robust locomotor ability after spinal cord transection, observed in elongated fish (eels) but lacking in amphibians and mammals. A recent modeling study (30) on lampreys suggested that stretch sensory feedback may be sufficient to recover swimming patterns when there is no descending activation of local CPG circuits (i.e., intrinsic frequencies of zero below transection), as long as the feedback gains are increased below the spinal transection (ten times higher than normal). Our results also confirm that increasing the feedback gains improves the chances of synchronization (Fig. 6*D*). Expanding from this hypothesis, we have explored the potential ability of multimodal sensory feedback in producing coordinated swimming, including the cases where local CPG circuits below spinal transection are capable of spontaneous oscillation. Specifically, we demonstrate that both pressure and stretch feedback immediately contribute to the self-organization of frequency-locked swimming and that having at least two of the three control components (central phase coupling, pressure feedback, stretch feedback) enables adaptation to the intrinsic frequency discrepancy between the anterior and posterior sections (Fig. 7). It should be noted that such coordinated swimming can be achieved even if sensory feedback gains are similar to those in the body section above the transection. Thus, our model suggests that spontaneous oscillations in the local segmental circuits and multimodal sensory feedback loops can be sufficient to entrain and coordinate the CPG rhythms along the body without assuming any additional (possibly time-consuming) adaptation in the neural system. Our work therefore provides a potential explanation why eels can recover locomotion immediately after a spinal transection (Fig. 6*A* and Movie S3), while other undulatory swimmers like salamanders require weeks before recovery (54). It is likely that animals like amphibians and mammals, which require time for nerve regeneration post spinal transection to recover locomotor function (54, 55), may be affected by their inability to generate spontaneous oscillation in local segmental circuits when descending drives are lost and/or by a lack of strength in their sensory feedback pathways.

## Materials and Methods

### Neuromechanical Simulation.

We constructed a two-dimensional model of the elongated fish in a horizontal plane, using the mass-spring-damper system (*SI Appendix*, Fig. S1). Specifically, we described the body as a chain of mass points serially connected via parallel combinations of a linear spring and a damper. To simulate the lateral body undulation, rotational actuators are implemented at each joint between body segments, and they generate torques according to the muscle model (46) derived from an antagonist muscle pair, approximated as linear springs and dampers, and adapted for rotational joints:

[6] $$\begin{array}{c} \tau_{i}=\alpha M_{i}-\gamma \theta_{i}-\delta {\dot{\theta }}_{i}. \end{array}$$

It generates a torque $\tau_{i}$ in the *i*th joint of the segmented body and contains the muscle activation $M_{i}$, the activation gain *α*, the stiffness *γ*, and the damping *δ*. The activation signal $M_{i}$ is produced by the segmental circuit (Eqs. **1**–**5**).

#### Physical Interactions Between the Body and Environment In Simulation.

In the simulated water environment, we assumed that hydrodynamic forces acting on the body are relative to the body’s speed. Similar to modeling work by Ekeberg (46), we assumed that inertial forces dominate during swimming (compared to viscous forces), and each mass point making up the body receives a pressure force proportional to the square of its velocity (*SI Appendix*, Fig. S2*A*). In the terrestrial environment, the simulated elongated fish was modeled under the assumption that its ventral surface remains in constant contact with the ground, with all mass points comprising the body subjected to Coulomb friction. To investigate the situation where propulsion on land cannot be achieved without reaction forces from pegs, the friction between the body and the ground was assumed to be isotropic. In addition, when the body contacts pegs, the mass point of the body segment receives a reaction force from the peg (*SI Appendix*, Fig. S2*B*). For a detailed description of the physical model, please refer to *SI Appendix*.

### Undulatory Robots to Validate Locomotor Ability in Aquatic and Terrestrial Environments.

Recognizing that simulations only approximate real-world physical dynamics, we conducted validations using an actual robot. This involved using AgnathaX, a robot inspired by lampreys, and directing it with our neuromechanical models to swim in a pool and traverse an array of pegs on the ground (Fig. 1*C*). Although AgnathaX and its exteroceptive sensors were initially developed primarily for swimming (22), we modified their design to enable the robot to navigate across an array of pegs on land. For a detailed explanation of the hardware design and experimental setup, please refer to *SI Appendix*.

### Parameter Selection for Simulation and Robot Experiments.

To verify that our proposed control circuits for locomotion function not only in simulations but also in real-world physics with robots, we selected physical and control parameters listed in Table S1 for each experiment. The physical parameters, such as mass, length scale, and muscle parameters, were taken from our previous study (22), which confirmed that the robot operates within a dynamic regime comparable to animals. To match the dynamics of simulations and the robot, we ensured these parameters were aligned to similar levels in both settings. For control parameters of segmental oscillators (Eq. **2**), we selected an intrinsic oscillator frequency of 1.5 Hz for swimming and 0.5 Hz for terrestrial crawling as representative levels based on behavioral data from animals (2, 25). However, due to hardware limitations, the actual robot experiments were conducted at half the frequencies used in the simulations (0.75 Hz for swimming and 0.25 Hz for terrestrial crawling). Specifically, the swimming frequency of the robot was reduced to keep power consumption within the theoretical limits of the servomotors (ensuring motor performance by operating them at an intermediate level) and to match the robot dynamics (such as the Strouhal number and Reynolds number) to levels observed in animals. Therefore, to ensure qualitative comparability between simulation and robot experiments despite differences in oscillator frequencies, we adjusted gain ranges for central phase coupling, pressure feedback, and stretch feedback ($\sigma_{c}$, $\sigma_{p}$, $\sigma_{s}$) in the robot experiments. Specifically, by setting these parameters to half the simulation levels, we maintained the relative magnitudes of sensory feedback and central phase coupling terms with respect to the intrinsic oscillator frequency.

### Performance Metrics.

#### Average frequency.

The average frequency of swimming $\overset{¯}{f}$ was measured by

[7] $$\begin{array}{c} \overset{¯}{f}=\frac{1}{N}\sum_{i=1}^{N}\frac{\phi_{i}\left(t_{\mathrm{end}}\right)-\phi_{i}\left(t_{\mathrm{start}}\right)}{2\pi (t_{\mathrm{end}}-t_{\mathrm{start}})}, \end{array}$$

where *N* is the number of body segment, and $t_{\mathrm{start}}$ and $t_{\mathrm{end}}$ are the start and end times of the measurement interval, respectively.

#### Overall phase lag.

The overall phase lag of swimming Φ(t) was measured by

[8] $$\bar{\phi }(t)=\arg\left(\sum_{j=1}^{N-1}e^{i(\phi_{j}(t)-\phi_{j+1}(t))}\right),$$

[9] $$\text{Φ}(t)=\frac{\bar{\phi }(t)\cdot (N-1)}{2\pi }\cdot 100,$$

where $\overset{¯}{\phi }(t)$ is the average intersegmental phase lag and *i* denotes the imaginary unit.

#### Transient time.

We defined the “transient time” as the time to reach the steady-state overall phase lag. For this purpose, the instantaneous overall phase lag Φ(t) was first smoothed with a moving average filter (1-s moving average), and then the transient time was measured as the time to reach within ±6% of the overall phase lag at the end time of measurement.

#### Stride length.

The stride length of swimming was calculated by multiplying the average CoM speed by the average cycle period ($1/\overset{¯}{f}$) over the measurement interval.

#### Mechanical CoT.

The mechanical CoT was measured by

[10] $$\begin{array}{c} \mathrm{CoT}=\frac{1}{\mathrm{mgd}}\int_{t_{\mathrm{start}}}^{t_{\mathrm{end}}}\sum_{i}\left|\tau_{i}{\dot{\theta }}_{i}\right|dt, \end{array}$$

where *m* is the total mass of the body, *g* is the gravitational acceleration, *d* is the distance traveled, and $\tau_{i}$ and ${\dot{\theta }}_{i}$ are the joint torque and angular velocity of the *i*th segment, respectively.

### Additional Simulation Experiments to Explore Different Topologies for the Stretch Feedback.

To explore possible different topologies for the stretch feedback, we additionally set two parameters in the stretch feedback term proposed in this study as below:

[11] $$\begin{array}{c} {\dot{\phi }}_{i}=2\pi f_{i}-\sigma_{s}\theta_{i+j}\sin\left(\phi_{i}+\chi \right). \end{array}$$

By modifying the subscript “*j*” of $\theta_{i+j}$, the location of the stretch sensor input referenced in the feedback (i.e., how many segments ahead or behind the sensory information comes from) can be adjusted. Additionally, by changing the parameter *χ*, which represents the phase shift, the adjustment timing of the oscillator phase in response to the stretch sensor input can be altered. Please see the simulation results in *SI Appendix*.

### Eel Transection.

*A. rostrata* (N=5) (length 314 ± 20 mm; mass 41.7 ± 9.0 g; mean ± SEM) swam in a standing water tank and filmed from above at 240 frames per second. Four electromyography (EMG) electrodes were implanted into the body muscle of the fish to record muscle activation at two different lengths down the fish (35.39 ± 1.24%, 61.64 ± 2.76%). After capturing normal swimming behavior, a spinal transection was performed at approximately 50% body length (50.01 ± 1.33%) and behavior was recorded again as soon as fish awoke from anesthetic. The body wave frequency was calculated at locations 25, 50, 75, and 100% down the body for both intact and transected fish. A representative trial was selected to visualize kinematic phase and muscle activation in a transected fish. The polar phase timing of the kinematic cycle was calculated at 10% increments along the body, with zero radians being a maximum left amplitude at that body location. EMG signals shown are from the right side of the fish and are normalized by the maximum EMG amplitude observed in the respective electrode to allow comparisons between electrodes.

## Supplementary Material

Appendix 01 (PDF)

Movie S1. Swimming using different controller types.

Movie S2. Terrestrial crawling on the ground with pegs using different controller types.

Movie S3. Swimming after spinal cord transection in eels and simulations using our model.

Movie S4. Swimming after spinal cord transection with the robot.

Movie S5. Effect of spatial-temporal shifts on the stretch feedback in our model.

## Data Availability

All study data are included in the article and/or supporting information.
