# Supplementary material for: Multisensory feedback makes swimming circuits robust against spinal transection and enables terrestrial crawling in elongate fish
Source: Proc Natl Acad Sci U S A. 2025 Aug 18;122(34):e2422248122. doi: 10.1073/pnas.2422248122 (PMC12403005; doi:10.1073/pnas.2422248122)
Supplement: Supplementary file 1 — Appendix 01 (PDF) [file pnas.2422248122.sapp.pdf]

Supporting Information for

Multi-sensory feedback makes swimming circuits robust against spinal transection and enables terrestrial crawling in elongate fish

Kotaro Yasui, Astha Gupta, Qiyuan Fu, Shura Suzuki, Jeffrey Hainer, Laura Paez, Keegan Lutek, Jonathan Arreguit, Takeshi Kano, Emily M. Standen, Auke J. Ijspeert, Akio Ishiguro

Kotaro Yasui, Astha Gupta
E-mail: kotaro.yasui.b8@tohoku.ac.jp, astha.gupta@epfl.ch

This PDF file includes:

Supporting text
Figs. S1 to S6
Table S1
Legends for Movies S1 to S5
SI References

Other supporting materials for this manuscript include the following:

Movies S1 to S5

Supporting Information Text

Model of physical interactions between the body and environment in simulation. In the simulated water environment, we assumed that the hydrodynamic forces acting on the body are relative to the body's speed. Similar to the modeling work by Ekeberg (1993) (1), we assumed that inertial forces dominate during swimming and each mass point of the body receives a resistive force proportional to the square of its velocity (Fig. S2A). Specifically, the normal component (F_i^{n}) and tangential component (F_i^{t}) of the resistive force acting on the ith body segment are described as:

$$|\boldsymbol{F}_i^{\mathrm{n}}| = \mu_{\mathrm{water,n}}|\boldsymbol{v}_i^{\mathrm{n}}|^2, \quad [1]$$

$$|\boldsymbol{F}_i^{\mathrm{t}}| = \mu_{\mathrm{water,t}}|\boldsymbol{v}_i^{\mathrm{t}}|^2, \quad [2]$$

where $\boldsymbol{v}_i^{\mathrm{n}}$ and $\boldsymbol{v}_i^{\mathrm{t}}$ are the normal and tangential components of the velocity of the ith body segment, respectively, and $\mu_{\mathrm{water,n}}$ and $\mu_{\mathrm{water,t}}$ are the drag coefficients.

In the terrestrial environment, the simulated elongated fish was modeled under the assumption that its ventral surface remains in constant contact with the ground, with all mass points of the body subjected to Coulomb friction as follows:

$$\boldsymbol{F}_i^{\mathrm{n}} = -\mu_{\mathrm{gnd,n}} m_i g \tanh(\rho_{\mathrm{gnd}}|\boldsymbol{v}_i^{\mathrm{n}}|)\boldsymbol{e}_i^{\mathrm{n}}, \quad [3]$$

$$\boldsymbol{F}_i^{\mathrm{t}} = -\mu_{\mathrm{gnd,t}} m_i g \tanh(\rho_{\mathrm{gnd}}|\boldsymbol{v}_i^{\mathrm{t}}|)\boldsymbol{e}_i^{\mathrm{t}}, \quad [4]$$

where $\boldsymbol{e}_i^{\mathrm{n}}$ and $\boldsymbol{e}_i^{\mathrm{t}}$ are the unit direction vectors corresponding to the normal and tangential direction of the ith body segment, respectively, and $\mu_{\mathrm{gnd,n}}$ and $\mu_{\mathrm{gnd,t}}$ are the friction coefficients, m_i denotes the mass and g is the gravitational acceleration constant and ρ is the positive constant. When $|\boldsymbol{v}_i^{\mathrm{n}}| \gg \rho_{\mathrm{gnd}}^{-1}$ and $|\boldsymbol{v}_i^{\mathrm{t}}| \gg \rho_{\mathrm{gnd}}^{-1}$, Eqs. 3 and 4 approximately describe Coulomb friction. In addition, when the body contacts a peg on the ground, the mass point of the body segment receives a reaction force from the peg (Fig. S2B). Specifically, we assumed a viscoelastic property of the interaction between the body and peg and that the mass point of the body segment receives the normal force from the peg ($F_i^{\mathrm{peg,n}}$) depending on the pushing amount. The pushing amount (d_i^{push}) is calculated by:

$$d_i^{\mathrm{push}} = r^{\mathrm{peg}} + r_i^{\mathrm{disc}} - d_i^{\mathrm{peg}}, \quad [5]$$

where r^{peg} is the radius of the peg, r_i^{disc} is the radius of the disc representing the body width, and d_i^{peg} is the distance between the centers of the peg and the body segment. Accordingly, the normal component ($F_i^{\mathrm{peg,n}}$) of the reaction force from the peg is described as:

$$\boldsymbol{F}_i^{\mathrm{peg,n}} = \max[k_{\mathrm{peg}} d_i^{\mathrm{push}} - c_{\mathrm{peg}} \boldsymbol{v}_i \cdot \boldsymbol{e}_i^{\mathrm{peg,n}}, 0]\boldsymbol{e}_i^{\mathrm{peg,n}}, \quad [6]$$

where k_{peg} is the spring constant, c_{peg} is the damping coefficient, and $\boldsymbol{e}_i^{\mathrm{peg,n}}$ is a unit direction vector that indicates the direction from the center of the peg to the center of the body segment. The tangential component ($F_i^{\mathrm{peg,t}}$) of the reaction force from the peg is modeled as Coulomb friction:

$$\boldsymbol{F}_i^{\mathrm{peg,t}} = -\mu_{\mathrm{peg}}|\boldsymbol{F}_i^{\mathrm{peg,n}}| \tanh(\rho_{\mathrm{peg}} \boldsymbol{v}_i \cdot \boldsymbol{e}_i^{\mathrm{peg,t}})\boldsymbol{e}_i^{\mathrm{peg,t}}, \quad [7]$$

where μ_{peg} is the friction coefficient, ρ_{peg} is the positive constant, and $\boldsymbol{e}_i^{\mathrm{peg,t}}$ is a unit direction vector that is orthogonal to the vector $\boldsymbol{e}_i^{\mathrm{peg,n}}$. When $|\boldsymbol{v}_i \cdot \boldsymbol{e}_i^{\mathrm{peg,t}}| \gg \rho_{\mathrm{peg}}^{-1}$, Eq. 7 approximately describes Coulomb friction.

In this study, for pressure force feedback (Eq. 4 in the main text), we used $\boldsymbol{F}_i^{\mathrm{n}}$ as the sensory input value ($\hat{F}_i$) during aquatic swimming and $\boldsymbol{F}_i^{\mathrm{peg,n}}$ during terrestrial crawling. Note that the magnitudes of these pressure sensor values were assumed to saturate at the threshold value F_{th}, which was set to 30 gf (0.29 N) both in simulations and robots. This was chosen based on the maximum force (30 gf) experienced by the robot during high-velocity swimming.

Robot experiments.

Hardware design. Our experiments took place in two distinct environments: swimming experiments in water and crawling experiments on land. We employed slightly modified versions of the robot tailored to meet the specific requirements of different experimental conditions. These adaptations ensured that the robot could be used reliably to assess the validity of simulation findings. The robot consisted of a head segment, 10 body segments, and a tail module. It measured 1.33 m in length and weighed 5.08 kg while swimming; during crawling, it was 1.27 m long and weighed 5.14 kg. The head module contained a Hardkernel Odroid XU4 computer running Linux, flanked by two lithium polymer (Li-Po) batteries. One of the batteries, rated at 7.4 V and 800 mAh, was used to supply power to the force sensors. The other, with a capacity of 11.1 V and 2200 mAh, powered the computer via a 5-V DC/DC regulator with a 25-W capacity. The placement of the batteries on either side of the computer was carefully adjusted to align the robot's center of mass with its centerline. Each body segment measured 90 mm in length. The head and body segments were serially linked via 10 Dynamixel MX-64 servomotors. Body segments were outfitted with a pair of force sensors on their lateral sides, customized specifically for either swimming or crawling experiments (for more details, see 'Exteroceptive Force Sensing'). The tail module was firmly attached to the last body segment. It comprised a rigid power unit and a flexible tail, both of which were uniquely designed to suit the requirements of the test environment. We reproduced extreme slipping conditions for crawling experiments using Teflon-lined skates. The Teflon skates were attached to the bottom of each module using a magnet. This resulted in a 0.142 static friction coefficient isometrically on the peg arena. These slipping conditions necessitated the use of obstacles for forward propulsion.

The robot (excluding the force sensors) was made waterproof for swimming tests with a two-layer fabric cover. The inner layer was a soft, hydrophobic, flexible sleeve enveloping the robot's modules. The outer layer was a specialized swimsuit crafted from yellow ripstop fabric with a thermo-adhesive coating for extra protection (KM-RoBoTa, Switzerland). The swimsuit was equipped with a waterproof zipper (MasterSeal 10, TIZIP, Germany) at the front for easy dressing of the robot. It had a waterproof 5-pole connector (693 series, Binder, Germany) integrated to enable communication between the computer and the external force sensors. During experiments, the servo motors were driven by an 11.1 V and 2200 mAh Li-Po battery, placed inside the power unit of the tail module. The compliant tail for swimming was 3-D printed using thermoplastic polyurethane (TPU) and measured 0.16 m long and 0.06 m tall (70 Shore A, 3 boundary layer, 40% infill). The tail-fin was attached to the tail module using magnets (Supermagnete, Switzerland) from outside the swimsuit. A set of stoppers was used to limit joint angles between -35 and 35 degrees, ensuring that self-collision between any two segments was avoided for the safety of the exteroceptive modules. The stoppers were fabricated using 3D printing with polylactic acid (PLA). A piece of cylindrical foam with a diameter of 60 mm and a height of 30 mm was magnetically secured to the top of each body segment. This modification rendered the robot slightly buoyant, enabling it to maintain surface-level swimming. Waterproofing layers were unnecessary and therefore not used during crawling experiments.

The tail module used during crawling contained a connector for tethered connection to an external 12V DC power supply. The tethered power supply was used during the experiments. The compliant tail-fin for crawling was stiffer than the swimming tail-fin (95 Shore A, all boundary layer). Stoppers, described above, were used during crawling to avoid self-collisions. Consequent to the absence of damping from water, the presence of obstacles, and a low friction environment, protection against collision forces from obstacles was essential. A rounded protective cover, made from PLA and installed on the head module, safeguarded the head. Moreover, modifications to the land exteroceptive sensing module were made to handle the destructive collision forces.

The Odroid computer runs control loops at 100 Hz in Linux. It collects proprioceptive data from the servomotors, including position, voltage, and current, and sends computed commands to the servomotors using the Dynamixel Protocol at 1 Mbps baud rate over RS-485 bus. The computer also collects exteroceptive data from the force sensors using the aforementioned protocol in a separate thread at 80 Hz. The latest value available in the memory was used for computation. We applied a low-pass filter through exponential smoothing to the force readings before integrating them as feedback. The response times (τ) of the filters were 94.9 ms for hydrodynamic forces and 4.3 ms for collision forces. This reduced the noise in sensor readings originating from the amplifier's excitatory high-frequency voltage ripples, waves of water reflected from the side of the pool walls while swimming, and vibrations of the mechanical parts (servo motors, screws, nuts, fasteners, etc.).

Exteroceptive Force Sensing. To enable exteroceptive sensing for feedback control, the robot was outfitted with two distinct sets of force sensors designed to detect hydrodynamic forces during swimming or collision forces during crawling, based on the design proposed by (2). The reason for the two different sets was to tune their sensitivity to their media, with small interaction forces in water, and much larger interaction forces with pegs (see below). Within each body segment, a pair of lateral force plates were utilized to assess the differential forces exerted on the body's left and right sides. The data from the two sensors on each segment were collected by two amplifiers (HX711, Sparkfun, US), respectively, and relayed to an Arduino Mini board positioned atop (crawling) or below (swimming) each module. Each Arduino board is integrated with a custom-made printed circuit board (PCB), featuring power management using a 5-V regulator and communication rerouting. These PCB boards are interconnected in a daisy-chain configuration and communicate with the head module through a custom serial communication protocol.

The set of hydrodynamic force sensors was designed to sit outside the suit and be exposed to water. Each force plate consists of load cells with a maximum measurable force of 300 gf (2.94 N; TAL221, HT Sensor Technology, China) attached to carbon fiber plates (60 mm × 44 mm × 2 mm). The integrated Arduino and PCB were waterproofed individually using a printed PLA box and a laser-cut polycarbonate lid sealed by a rubber O-ring. The amplifiers were waterproofed using Sicomin GreenPoxy 56 (Swiss-Composite). The set of collision force sensors used load cells with a maximum measurable force of 5 kgf (49 N; TAL220A, HT Sensor Technology, China). The load cells were attached to 3D-printed polycarbonate force plates (59 mm × 48 mm). These plates allowed a movement range of -35 to 35 degrees. The exposed surfaces of the force plates were covered with Teflon-coated tapes to reduce the friction between the plates and the pegs. Furthermore, 3-D printed PLA covers and TPU cushions were used to protect the amplifiers against the shock from collision.

For the pressure force feedback control, we used a binary threshold saturation protocol for the robot crawling experiments, since the forces measured in water and on the ground differ by two orders of magnitude. Specifically, the saturation value of 30 gf (0.29 N) was applied when the force measured was greater than the threshold value of 50 gf (0.49 N, 1% of the load cell range), otherwise, the force value was set to 0 gf (0.0 N). This saturation threshold was chosen to avoid ghost saturation due to sensor noise.

Experimental Setup. The experimental setup consisted of the arena and tracking system. Each arena was tracked with a GoPro 11 in 4K 60fps video mode. The GoPro was mounted to have a top view of the experimental arena. To account for slight differences in the mounting angle across trials, the corners of the arenas were marked with colored tapes and tracked for correction. Swimming tests were conducted in a 6 m × 2 m pool. We used a wide-angle mode to capture the full length of the pool, which was later undistorted through camera calibration methods of OpenCV. During swimming, the 2-D positions of the 10 body segments were tracked using LED lights mounted on top of the segments. Crawling experiments were conducted in a peg arena which consisted of six horizontal wooden pallets fastened and leveled together. The arena measured 2.4 m by 1.8

m and featured 7 × 6 grid of obstacles placed 0.31 m apart from center to center. The obstacles were cylindrical in shape, with 110 mm in diameter and 104 mm in height. The peg arena was tracked in the linear mode of the GoPro camera. The 2-D positions of the robot segments were tracked using custom multicolor markers(35 mm × 30 mm) with black squares at the centers(5 mm × 5 mm). We used a custom algorithm in OpenCV to track LEDs for swimming experiments and colored markers for crawling experiments.

Additional simulation experiments to explore different topologies for the stretch feedback. To explore possible different topologies for the stretch feedback, we additionally set two parameters in the stretch feedback term proposed in this study as below:

$$\dot{\phi}_i = 2\pi f_i - \sigma_s \theta_{i+j} \sin(\phi_i + \chi). \qquad [8]$$

By modifying the subscript 'j' of θ_{i+j}, the location of the stretch sensor input referenced in the feedback (*i.e.*, how many segments ahead or behind the sensory information comes from) can be adjusted. Additionally, by changing the parameter χ, which represents the phase shift, the adjustment timing of the oscillator phase in response to the stretch sensor input can be altered. To investigate the pure effect of the change in the topology of stretch feedback, here we used a decoupled oscillator with only stretch feedback (Eq. 8). Tested control parameters are $f_i = 1.5$[Hz]$, \sigma_{\mathrm{s}} = 20$[1/s], and 20 trials were performed for each experiment with randomly set initial oscillator phases.

By varying 'j' from -2 to 1 under $\chi = 0$ (Fig. S3A-B, Movie S5), we first found that our proposed feedback topology ($j = -1$) generated the most effective forward swimming (*i.e.*, the highest stride length). In contrast, when the stretch of the same segment ($j = 0$) or the stretch of the segment two ahead ($j = -2$) was referenced, the resulting pattern varied according to the initial phase conditions, and a phase difference close to π (*i.e.*, almost anti-phase) between adjacent oscillators was often observed, which prevented effective forward propulsion. Furthermore, when the stretch of segment one behind was referenced ($j = 1$), the body converged to a traveling wave pattern propagating from tail to head, leading to coordinated backward swimming.

Since the resulting swimming pattern can be changed according to the combinations of spatial shift (j) and temporal shift (χ) in the sensory feedback topology, we next tested the change in the phase shift parameter χ from $-\frac{\pi}{2}$ to $\frac{\pi}{2}$under the cases of $j = \{-1, 1\}$ (Fig. S3C-D, Movie S5). In the case of $j = -1$, we found that our proposed feedback topology (temporal shift of $\chi = 0$) exhibited almost the highest performance in terms of stride length and the shortest transient time to the steady state regime for pattern generation. Interestingly, in the case of $j = 1$ (Fig. S3D), when the temporal phase shift (χ) was between $-\frac{\pi}{2}$ and $-\frac{\pi}{4}$, it exhibited similarly high swimming performance (stride length, transient time) as our proposed feedback ($j = -1$, $\chi = 0$). Thus, the feedback topology of $j = -1$ and $\chi = [-\frac{\pi}{2}, -\frac{\pi}{4}]$ could be another possible circuit useful for swimming.

Finally, we tested whether the abovementioned feedback that references the stretch of the segment one behind ($j = 1$ and $\chi = [-\frac{\pi}{2}, -\frac{\pi}{4}]$), which showed high performance in swimming, also functions during crawling on the ground with pegs. The experimental setup and parameters are the same as in the experiments in the main text (Fig. 4). Figure S4 shows the rate of successful traversal, and we found that this stretch feedback topology does not work effectively in dry ground locomotion, including the cases when it was combined with central phase coupling (C) and pressure feedback (P).

Robot experiments to test the robust swimming ability after spinal cord transection using our model. We conducted swimming experiments with the robot to validate our findings in the simulation experiments shown in Fig. 6D. The spinal transection was performed using our model as described in Fig. 6C. Note that in these robot experiments, the servo motor between the 5th and 6th segment (*i.e.*, the spinal-transected part) was actively controlled, which was different from the simulation experiment where the corresponding muscle was set to be passive. Here, the intrinsic oscillator frequency for the segments above the transection was set to be 0.75 Hz, and only the controller type with pressure and stretch feedback (type P+S) was tested. Feedback gains for pressure and stretch were $\sigma_p = 100, \sigma_s = 15$, respectively. Figure S6 shows the synchronization level of the swimming frequencies between the segments above/below the spinal transection. The frequency-locked synchronized swimming was observed when the intrinsic oscillator frequency below the transection (1.0 Hz) was equal or 33% higher than that of the anterior segments (see the point marked with a star in Fig. S6). In contrast, it did not emerge when the intrinsic oscillator frequency below the transection was lower or even higher. The results demonstrated similar characteristics to those found in the simulation experiments.

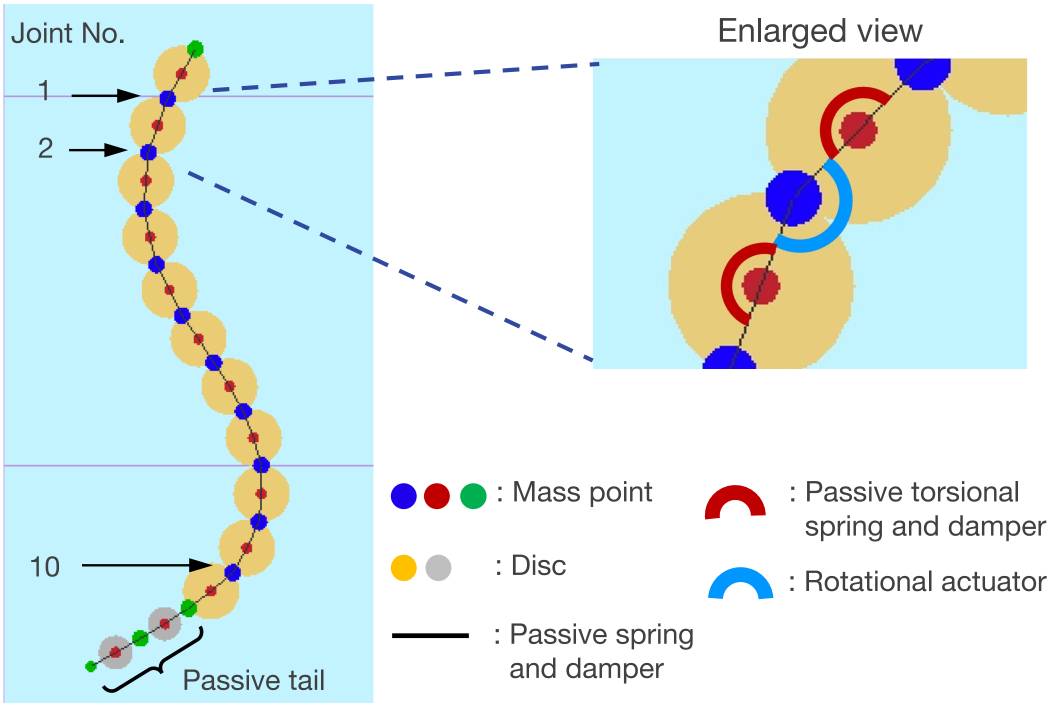

Fig. S1. Schematic of the simulated model of elongated fish. Note that yellow and gray discs describe the body width and are only used in the terrestrial environment to judge the body contact with the pegs and calculate the reaction force from the pegs. The blue rotational actuators between the segments generate torque based on the muscle model.

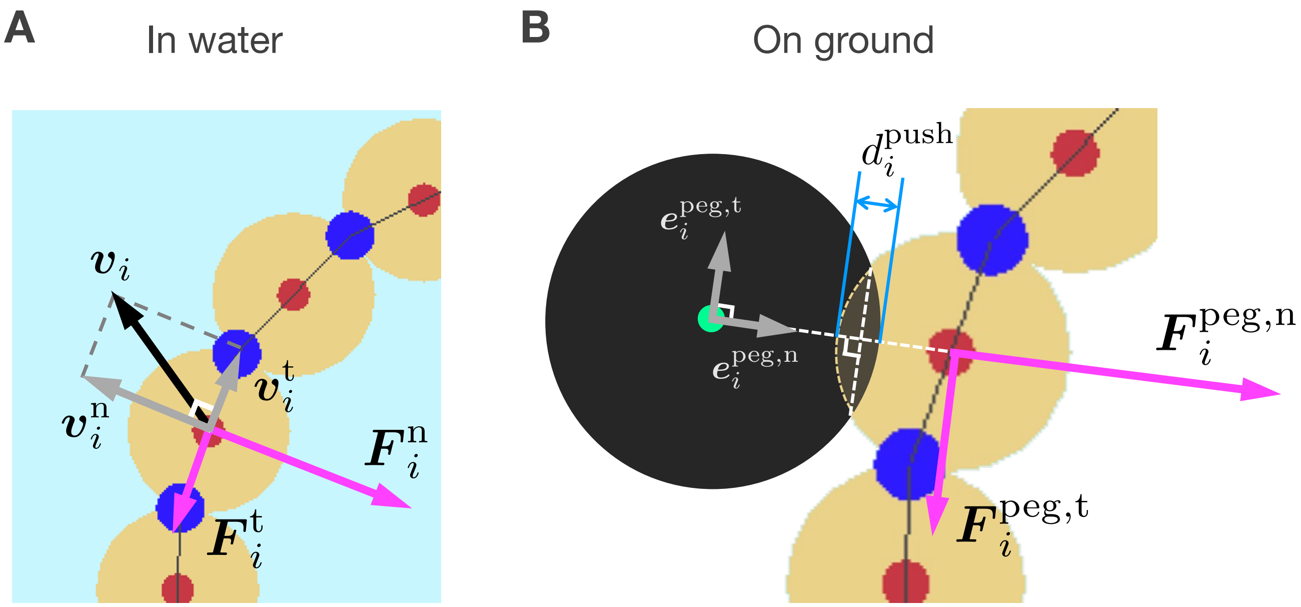

Fig. S2. Schematic of the physical interactions between the body and environment in simulations. (A) In water, all the mass points along the body receive resistive forces proportional to the square of its velocity. (B) On the ground with pegs, when the body segment (represented by a yellow disc) makes contact with a peg (black circle), it receives the normal force ($F_i^{\mathrm{peg,n}}$) and tangential friction force ($F_i^{\mathrm{peg,t}}$) from the peg. Green circle indicates the center of the peg and d_i^{push} represents the pushing amount of the body against the peg.

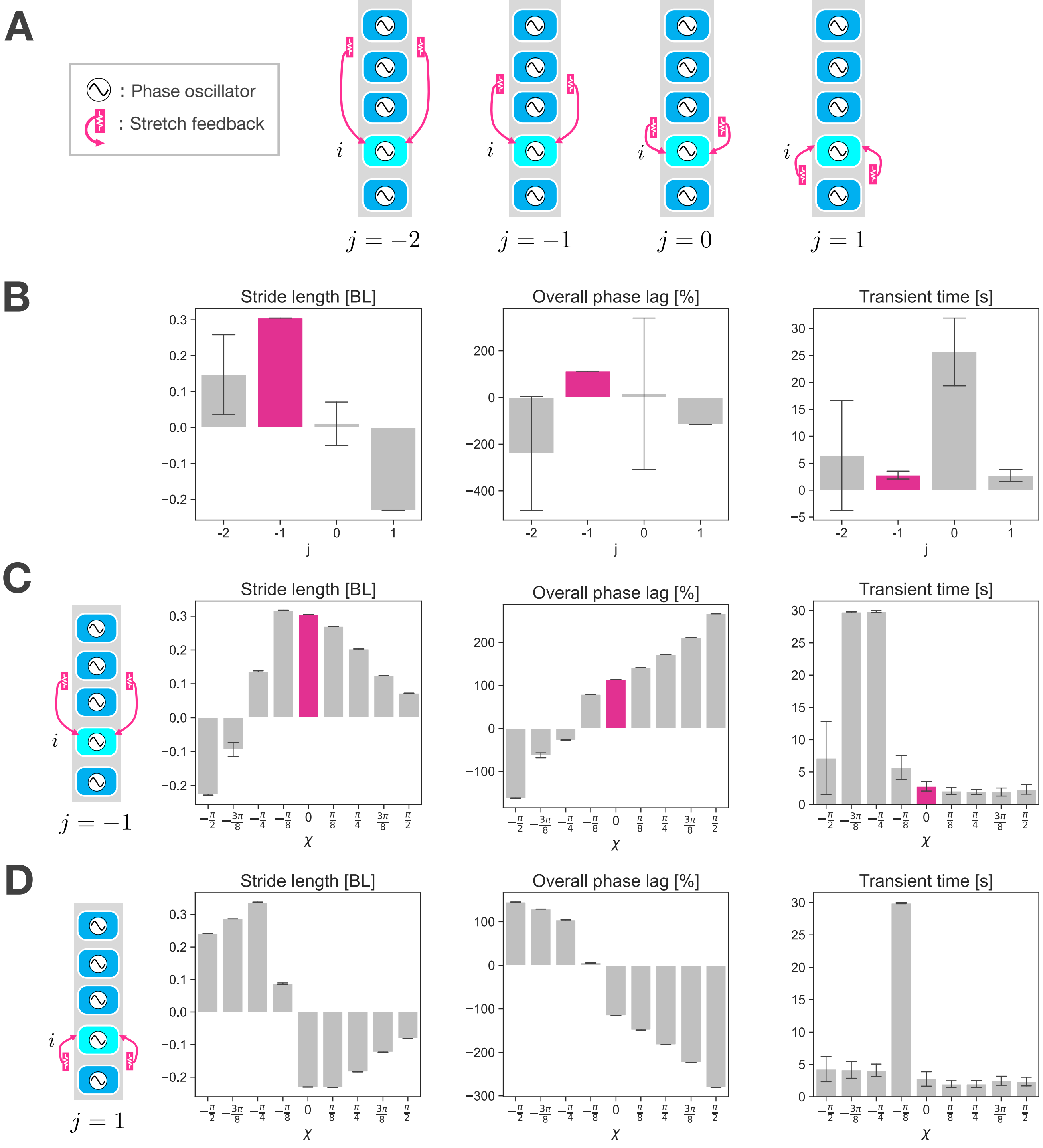

Fig. S3. Effect of changes in the topology of the stretch feedback on swimming pattern generation. (A) Schematic of the tested spatial shifts in the sensory feedback. (B-D) Stride length (left panels) and overall phase lag (middle panels) of the swimming and transient time to reach the steady state swimming (right panels) were evaluated when (B) stretch sensor inputs from different body sections were fed back to decoupled segmental oscillators (*i.e.*, spatial shifts of the feedback connection) in cases of $\chi = 0$, and when (C, D) the parameter χ for the phase shift was varied for the cases where the spatial shifts are $j = -1$ and $j = 1$, respectively. The proposed stretch feedback topology in this study is highlighted in magenta color. The error bars indicate the standard deviation.

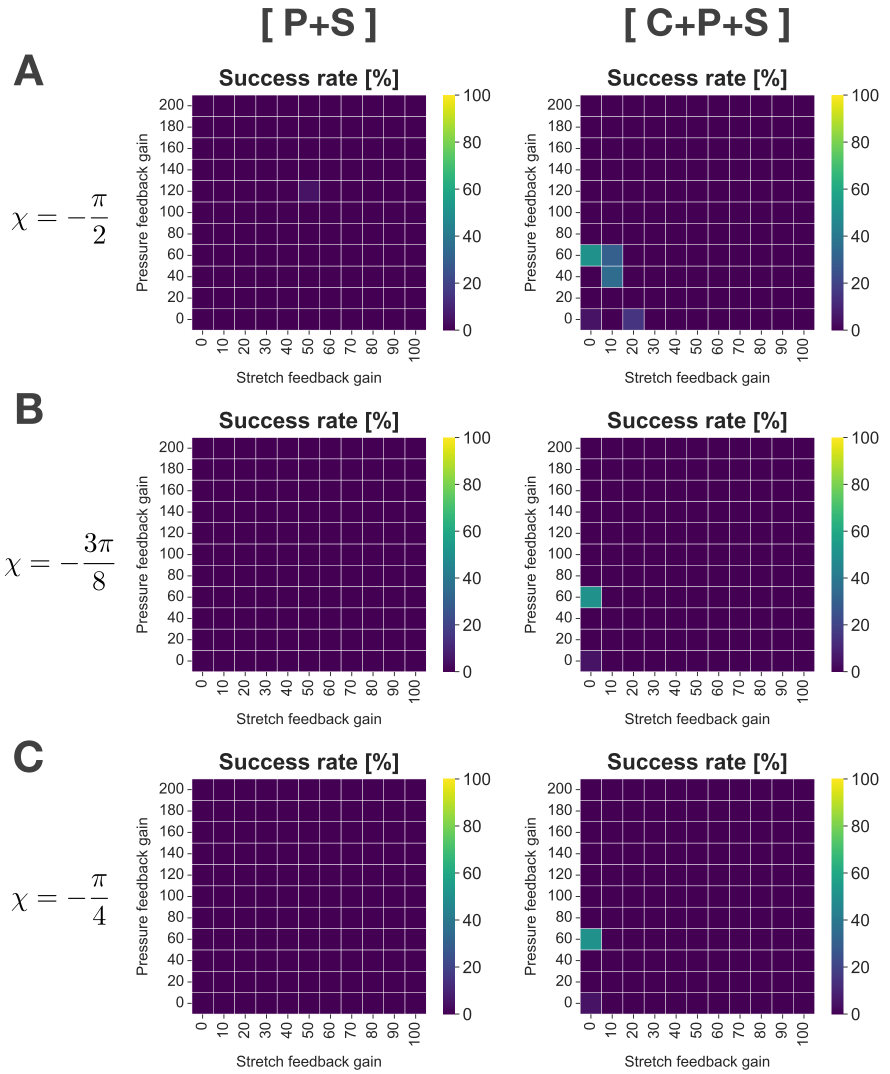

Fig. S4. Rate of successful traversal on the ground with pegs using the stretch feedback which refers to the sensory input at one segment behind (spatial shift parameter $j = 1$). (A)-(C) represent the simulation results when the phase shift parameters are $\chi = -\frac{\pi}{2}, -\frac{3\pi}{8}, -\frac{\pi}{4}$, respectively.

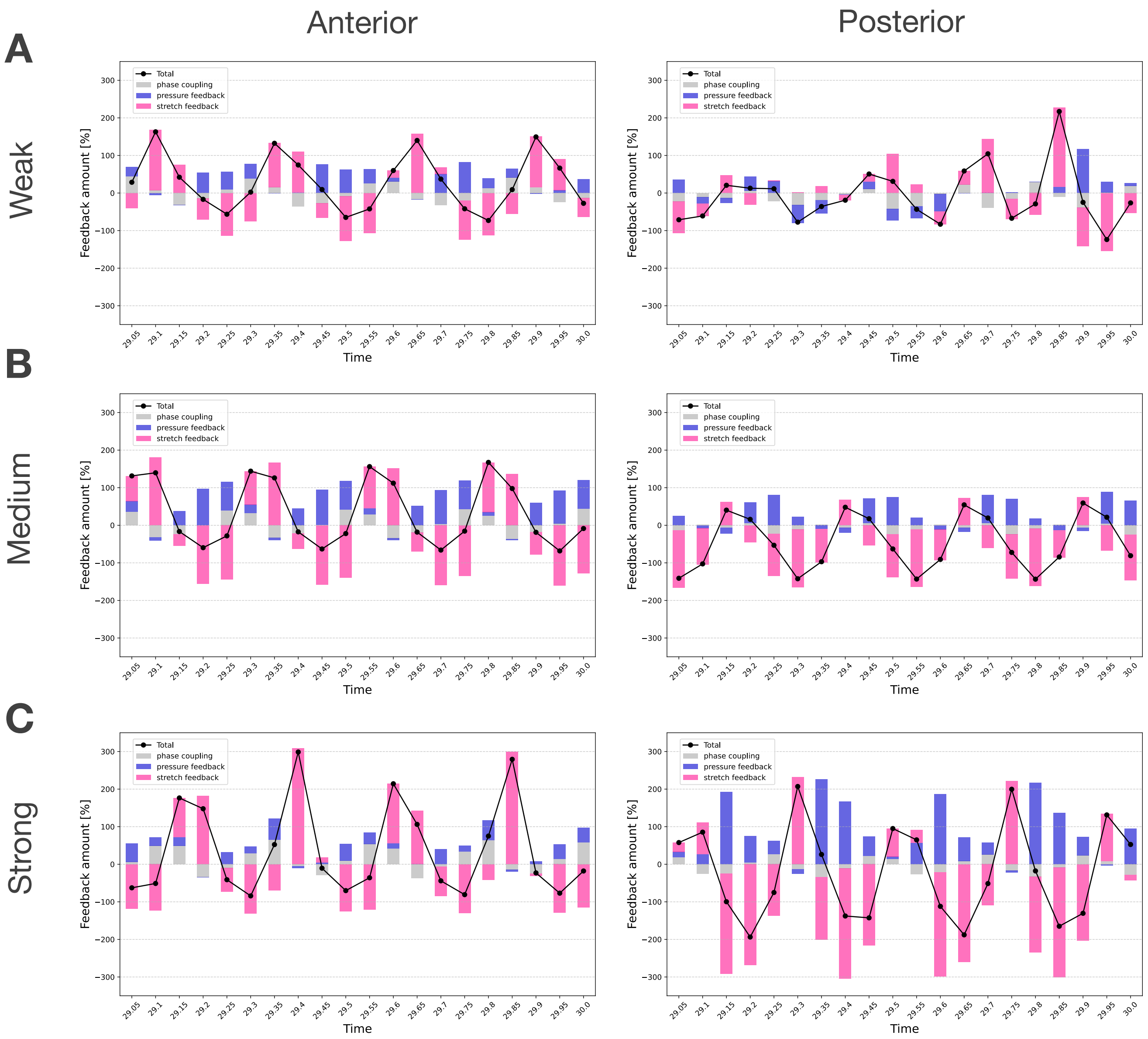

Fig. S5. The relative contribution of central phase coupling and sensory feedbacks to oscillator phase modulation in the spinal transected simulation. The panels in the left column show data for the oscillator in the anterior body segment (3rd), while the panels in the right column show data for the oscillator in the posterior body segment (8th) below the transection. Each row corresponds to cases where the gain of stretch and pressure feedback is (A) weak, (B) medium, and (C) strong, as shown in Fig. 6D. The data presented here is the result when an intrinsic oscillator frequency was 2.7 Hz in the posterior segment, which was 1.8 times higher than that of the anterior segment (1.5 Hz). The bar graphs represent the feedback amount of each control term in Eq. 2, normalized by the intrinsic phase velocity of the oscillators in the anterior segment (*i.e.*, $2\pi f_i$ in Eq. 2). The black dot indicates the net feedback amount, obtained by summing all feedback contributions. Positive feedback values accelerate the oscillator phase, while negative values decelerate it.

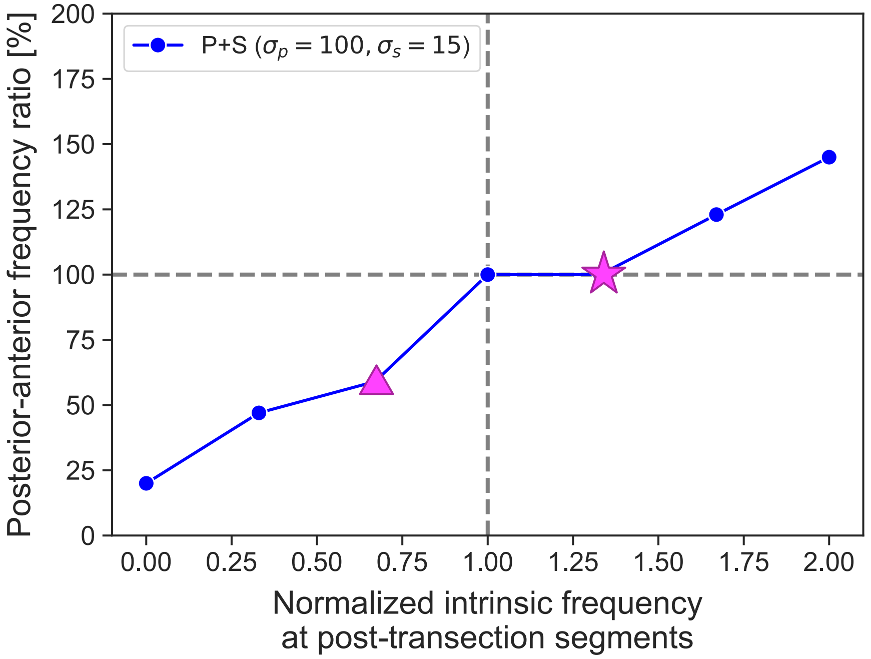

Fig. S6. Synchronization level of the swimming frequencies between the segments above/below the spinal transection when the controller type P+S was implemented with the robot. Triangle and star marks correspond to those shown in Fig. 6D in the main text and to the two swimming sequences shown in Movie S4. Experimental details are described in the Supporting Text.

Table S1. Parameter values employed in simulation and robot experiments

	Simulation		Robot	
	Value	Unit	Value	Unit
Simulation time step	0.0005	[s]		
N (number of segments)	10		10	
Total body mass	4.34	[kg]	(swim) 5.08	[kg]
			(crawl) 5.14	[kg]
Head mass	0.455	[kg]		
Segment mass	0.35	[kg]		
Tail mass	0.385	[kg]		
Total body length	1.17	[m]	(swim) 1.33	[m]
			(crawl) 1.27	[m]
Head length	0.09	[m]		
Segment length	0.09	[m]	0.09	[m]
Segment width (disc diameter)	0.09	[m]		
Tail length	0.18	[m]		
α (activation gain)	0.9	[Nm]	0.9	[Nm]
γ (muscle stiffness)	1.7	[Nm/rad]	1.6	[Nm/rad]
δ (muscle damping)	0.05	[Nm/rad]	0.0	[Nm/rad]
Tail stiffness	1.5	[Nm/rad]		
Tail damping	0.02	[Nms/rad]		
ψ (central phase lag bias)	$2\pi/N$	[rad]	$2\pi/N$	[rad]
$\mu_{\mathrm{water,n}}$	3.11	$[\mathrm{Ns^2/m^2}]$		
$\mu_{\mathrm{water,t}}$	0.155	$[\mathrm{Ns^2/m^2}]$		
$\mu_{\mathrm{gnd,n}}$	0.05			
$\mu_{\mathrm{gnd,t}}$	0.05			
ρ_{gnd}	1.0			
r_{peg} (peg radius)	0.054	[m]	0.055	[m]
Distance between the centers of neighboring pegs	0.306	[m]	0.31	[m]
k_{peg}	1050	[N/m]		
c_{peg}	17.5	[Ns/m]		
μ_{peg}	0.05			
ρ_{peg}	0.1			
Swimming experiments in Figs. 2 and 3				
f_i	1.5	[Hz]	0.75	[Hz]
σ_c	5	[rad/s]	2.5	[rad/s]
σ_p	40	[rad/Ns]	100	[rad/Ns]
σ_s (Fig. 2)	60	[1/s]	15	[1/s]
σ_s (Fig. 3)	0-120	[1/s]		
Crawling experiments in Figs. 4 and 5				
f_i	0.5	[Hz]	0.25	[Hz]
σ_c	5	[rad/s]	2.5	[rad/s]
σ_p (Fig. 4)	40	[rad/Ns]	8.33	[rad/Ns]
σ_s (Fig. 4)	60	[1/s]	25	[1/s]
σ_p (Fig. 5)	0-200	[rad/Ns]	0-100	[rad/Ns]
σ_s (Fig. 5)	0-100	[1/s]	0-50	[1/s]
Transected swimming experiments in Figs. 6 and 7				
σ_c	5	[rad/s]		
σ_p (Weak-Medium-Strong, in Fig. 6D)	40-60-80	[rad/Ns]		
σ_s (Weak-Medium-Strong, in Fig. 6D)	60-90-120	[1/s]		
σ_p (Fig. 7)	60	[rad/Ns]		
σ_s (Fig. 7)	90	[1/s]		
Spatial-temporal shift experiments in Figs. S3 and S4				
f_i	1.5	[Hz]		
σ_s (Fig. S3)	20	[1/s]		
σ_c (Fig. S4)	5	[rad/s]		
σ_p (Fig. S4)	0-200	[rad/Ns]		
σ_s (Fig. S4)	0-100	[1/s]		

Movie S1. Swimming using different controller types.

Movie S2. Terrestrial crawling on the ground with pegs using different controller types.

Movie S3. Swimming after spinal cord transection in eels and simulations using our model.

Movie S4. Swimming after spinal cord transection with the robot.

Movie S5. Effect of spatial-temporal shifts on the stretch feedback in our model.

References

1. Ö Ekeberg, A combined neuronal and mechanical model of fish swimming. *Biol. cybernetics* **69**, 363–374 (1993).
2. R Thandiackal, et al., Emergence of robust self-organized undulatory swimming based on local hydrodynamic force sensing. *Sci. robotics* **6**, eabf6354 (2021).
